# Supplementary material for: Rhodanine-based Knoevenagel reaction and ring-opening polymerization for efficiently constructing multicyclic polymers
Source: Nat Commun. 2020 Jul 21;11:3654. doi: 10.1038/s41467-020-17474-0 (PMC7374721; doi:10.1038/s41467-020-17474-0)
Supplement: Supplementary file 1 — Supplementary Information [file 41467_2020_17474_MOESM1_ESM.pdf]

Supplementary Information

Zhang et al.

Rhodanine-based Knoevenagel Reaction and Ring-Opening Polymerization for Efficiently Constructing Multicyclic Polymers

Ze Zhang[1], Xuan Nie[1], Fei Wang[2], Guang Chen[1], Wei-Qiang Huang[1], Xia Lei[1], Wen-Jian Zhang[1], Zong-Yao Hao[3*], Chun-Yan Hong[1*], Long-Hai Wang[1*] & Ye-Zi You[1*]

[1]CAS Key Laboratory of Soft Matter Chemistry, Department of Polymer Science and Engineering, University of Science and Technology of China, Hefei, Anhui 230026, People's Republic of China.
[2]The First Affiliated Hospital of University of Science and Technology of China, Hefei, Anhui 230001, People's Republic of China.
[3]the First Affiliated Hospital of Anhui Medical University and Institute of Urology, Anhui Medical University, Hefei, Anhui 230022, People's Republic of China.

Content

Supplementary Methods....4
General Reagent Information....4
General Analytical Information....5
Synthesis of 2-(phenoxymethyl) thiirane (POMT).....6
Synthesis of 2-((prop-2-yn-1-yloxy)methyl)thiirane (PYMT).....6
Synthesis of 2-((allyloxy)methyl)thiirane (PEMT).....6
Synthesis of 5-(4-(diphenylamino)phenyl)thiophene-2-carbaldehyde (TTPA).....7
Synthesis of bifunctional rhodanine monomer.....7
Synthesis of rhodanine-containing methyl methacrylate (MRDA).....8
Model reaction of rhodanine-aldehyde condensation....9
Ring-opening polymerization of thiiranes using rhodanine and derivatives as initiators....9
Synthesis of cyclic brush-like polymer....10
Synthesis of multicyclic polymers with cyclic units in the backbone.....10
Synthesis of red/near-infrared AIE multicyclic polymer with pendant cyclic units.....11
Supplementary Figure....12

Supplementary Figure 1. ^{1}H NMR trace during the formation of cyclic polymer: Conversion (POMT) = (1-I_a) × 100%; I_a denotes the protons integral values of methylene at unreacted POMT (δ = 4.00 ppm)....13
Supplementary Figure 2. ^{1}H NMR trace without adding rhodanine as the initiator....14
Supplementary Figure 3. The ^{1}H NMR spectrum of the cyclic polymer in entry 1, Table 115
Supplementary Figure 4. ^{13}C NMR spectrum of the cyclic polymer in entry 1, Table1 15
Supplementary Figure 5. The ^{1}H NMR spectrum of the cyclic polymer in entry 2, Table 116
Supplementary Figure 6. The MALDI-TOF MS spectrum of the cyclic PPYMT in entry 2, Table 1....16
Supplementary Figure 7. The ^{1}H NMR spectrum of the cyclic polymer in entry 3, Table 1 using *N*-substituent rhodanine derivative as the initiator....17
Supplementary Figure 8. SEC curve of resulting polymer in entry 3, Table 1 using *N*-substituent rhodanine derivative as the initiator....17
Supplementary Figure 9. MALDI-TOF MS spectrum of resulting polymer in entry 3, Table 1 using *N*-substituent rhodanine derivative as the initiator....18
Supplementary Figure 10. The ^{1}H NMR spectrum of cyclic random copolymer in entry 4, Table 1....18
Supplementary Figure 11. The ^{1}H NMR spectrum of the cyclic graft copolymer....19
Supplementary Figure 12. The SEC curves of the cyclic copolymer and cyclic graft copolymer....19
Supplementary Figure 13. The mechanism of RA Knoevenagel reaction....20
Supplementary Figure 14. The HR-MS analysis of RA reaction in entry 1, Table 2....20
Supplementary Figure 15. The ^{1}H NMR trace of RA reaction in entry 2, Table 2....21
Supplementary Figure 16. The ^{13}C NMR trace of RA reaction in entry 2, Table 2....21
Supplementary Figure 17. The ^{1}H NMR trace of RA reaction in entry 3, Table 2....22
Supplementary Figure 18. The ^{13}C NMR trace of RA reaction in entry 3, Table 2....22
Supplementary Figure 19. The ^{1}H NMR trace of RA reaction in entry 4, Table 2....23

Supplementary Figure 20. The ^{13}C NMR trace of RA reaction in entry 4, Table 2..............23
Supplementary Figure 21. The ^{1}H NMR trace of RA reaction in entry 5, Table 2...............24
Supplementary Figure 22. The ^{13}C NMR trace of RA reaction in entry 5, Table 2..............24
Supplementary Figure 23. The ^{1}H NMR trace of RA reaction in entry 6, Table 2...............25
Supplementary Figure 24. The ^{13}C NMR trace of RA reaction in entry 6, Table 2..............25
Supplementary Figure 25. The ^{1}H NMR trace of RA reaction in entry 7, Table 2...............26
Supplementary Figure 26. The ^{13}C NMR trace of RA reaction in entry 7, Table 2..............26
Supplementary Figure 27. The ^{1}H NMR trace of RA reaction in entry 8, Table 2...............27
Supplementary Figure 28. The ^{13}C NMR trace of RA reaction in entry 8, Table 2..............27
Supplementary Figure 29. The HR-MS analysis of RA reaction in entry 2, Table 2 28
Supplementary Figure 30. The HR-MS analysis of RA reaction in entry 3, Table 2 28
Supplementary Figure 31. The HR-MS analysis of RA reaction in entry 4, Table 2 29
Supplementary Figure 32. The HR-MS analysis of RA reaction in entry 5, Table 2 29
Supplementary Figure 33. The HR-MS analysis of RA reaction in entry 6, Table 2 30
Supplementary Figure 34. SEC curve of resulting polymer using *C*-substituent RA reaction product in Figure 4 as the initiator..30
Supplementary Figure 35. MALDI-TOF MS spectrum of resulting polymer using *C*-substituent RA reaction product in Figure 4 as the initiator...31
Supplementary Figure 36. ^{13}C NMR spectrum of RA Knoevenagel polymerization product and corresponding monomers 31
Supplementary Figure 37. ^{1}H NMR spectrum of mixture during the formation of multicyclic copolymer: Conversion (POMT) = (1-I_a) × 100%; I_a denotes the protons integral values of methylene at unreacted POMT (δ = 4.00 ppm)..32
Supplementary Figure 38. The ^{1}H NMR spectra of the multicyclic copolymer with cyclic units in the backbone at different conversions...32
Supplementary Figure 39. ^{13}C NMR analysis of the precursor polymer and multicyclic polymers..33
Supplementary Figure 40. TGA of the precursor polymer and multicyclic polymers 33
Supplementary Figure 41. DSC curves of multicyclic polymers and single cyclic counterpart 34
Supplementary Figure 42. TEM image of multicyclic copolymer brush 34
Supplementary Figure 43. SEC curves of resulting hyperbranched polyrhodanine initiator and hyperbranched multicyclic polymers 35
Supplementary Figure 44. ^{1}H NMR spectra of resulting hyperbranched polyrhodanine initiator and hyperbranched multicyclic copolymer..35
Supplementary Figure 45. ^{1}H NMR spectrum of rhodanine-containing methyl methacrylate (MRDA)..36
Supplementary Figure 46. UV−vis characteristic absorption curves of trithiocarbonate containing copolymer 1 and copolymer 2 in Figure 8...36
Supplementary Figure 47. The typical donor-acceptor structure of RA reaction product....37
Supplementary Figure 48. The ^{1}H NMR spectra of copolymer 2 with side rhodanine units and red/near-infrared AIE copolymer 3 with side rhodanine units 37
Supplementary Figure 49. The ^{1}H NMR spectrum of red/near-infrared AIE multicyclic polymer 4 with pendant cyclic units 38

Supplementary Methods

General Reagent Information

All reagents were used as received unless otherwise stated. Glycidyl propargyl ether (90%), PEG-OH (M_n = 2000 g/mol) and 4-dimethylaminopyridine (98%) were purchased from Aladdin. Rhodanine (99%), rhodanine-3-acetic acid (98%), 2-(phenoxymethyl) oxirane (98%), 5-formyl-2-thiopheneboronic acid (98%), tetrakis(triphenylphosphine)palladium (99%), 2,4,6-Trimethylbenzaldehyde (98%) and pentaethylene glycol (97%) were purchased from Energy Chemical. 4-Bromotriphenylamine (99%), 4-nitrobenzaldehyde (99%), 4-(hydroxymethyl)benzaldehyde (99%), 4-ethynylbenzaldehyde (98%), terephthalaldehyde (99%) and tetrabutylammonium chloride (99%) were purchased from Alfa Aesar. 4-Acetamidobenzenesulfonyl azide (97%) was purchased from Adamas-Beta. Chloroform-d (99.8 atom % D), deuterium oxide (99.9 atom % D), MECN-d_3 (99.8 atom % D), DMSO-d_6 (99.8 atom % D) and DMF-d_7 (99.5 atom % D) were purchased from Sigma-Aldrich. 3-Allylrhodanine (98%), 2-hydroxyethyl methacrylate (95%), furfural (98%) and dicyclohexylcarbodiimide (98%) were purchased from TCI. Methyl methacrylate (99.8%), sodium sulfate anhydrous (99%), potassium thiocyanate (99%), n-hexane (97%), ethyl acetate (99.5%), methanol (99.7%), chloroform (99%), acetone (99.5%), diethyl ether (99.7%) and cuprous chloride (98.5%) were purchased from Sinopharm Chemical Reagent Co. Ltd. Methyl methacrylate was purified by small aluminum oxide (basic) chromatography to remove inhibitor.

General Analytical Information

All NMR spectra were recorded on a Bruker NMR spectrometer (resonance frequency of 400 MHz for ^1H and 100 MHz for ^{13}C) operated in the Fourier transform mode. The samples were dissolved in deuterium oxide, chloroform-d, MECN-d_3, DMF-d_7 or DMSO-d6 with tetramethylsilane (TMS) as an internal reference. Molecular weights and dispersity (*Đ*) were measured by using a Waters 150C gel permeation chromatograph (GPC) equipped with microstyragel columns and an RI 2414 detector at 30 °C. LiBr/DMF (0.1%, w/w) solution or THF solution with a flow rate of 1.0 mL/min were used as eluent. The molecular weights were calibrated against monodispersed polystyrene standards. High resolution mass spectrometry data were obtained from ThermoFisher LTQ-Orbitrap XL instrument. MALDI-TOF/TOF spectra were obtained using Auto Flex II mass spectrometer (Bruker Daltonics), and DCTB was used as a matrix. Imaging of the cyclic brush-like polymer and multicyclic molecular brush were accomplished using Bruker atomic force microscope system in ambient air. Thermogravimetric analysis was measured on a TA Q5000IR instrument with a heating rate of 10 °C/min from room temperature to 700 °C. Differential scanning calorimetry thermograms were measured on a TA Q2000 differential scanning calorimeter instrument in aluminum pans with a heating or cooling rate of 10 °C/min under a flowing nitrogen atmosphere from -40 °C to 120 °C. All T_g values were obtained from the second scan after removing the thermal history. PL spectra were obtained from Hitachi F-7000 fluorescence spectrophotometer. The absolute fluorescence quantum yields of copolymer 3 solid and multicyclic copolymer 4 solid were measured by FluoroMax-4 steady state and transient fluorescence spectrometer with integrating sphere. TEM (Hitachi 7700), HRTEM (JEOL JEM 2100 plus) and AFM (Bruker edge) were used to image the cyclic structures.

Synthesis of 2-(phenoxymethyl) thiirane (POMT).

To a suspension of 2-(phenoxymethyl)oxirane (9.0 g, 60 mmol) in distilled water (30 mL), was added potassium thiocyanate (23.2 g, 240 mmol) and stirred at 40 °C for 24 h. Afterwards, the organic phase was separated and the aqueous phase was extracted with diethyl ether (2 × 30 mL). The combined organic phases were dried over anhydrous sodium sulfate. Further purification can be achieved by silica gel column chromatography using hexane/ethyl acetate (v/v, 9 : 1) to obtain the product as colorless viscous oil. Yield was 75%. ^{1}H NMR spectrum (400 MHz, $CDCl_3$): δ 2.349 (d, 1H), δ 2.632 (d, 1H), δ 3.307 (m, 1H), δ 3.961 (m, 1H), δ 4.224 (m, 1H), δ 6.959 (m, 3H), δ 7.325 (m, 2H).

Synthesis of 2-((prop-2-yn-1-yloxy)methyl)thiirane (PYMT).

To a suspension of 2-((prop-2-yn-1-yloxy)methyl)oxirane (6.72 g, 60 mmol) in distilled water (30 mL), was added potassium thiocyanate (23.2 g, 240 mmol) and stirred at 40 °C for 24 h. Afterwards, the organic phase was separated and the aqueous phase was extracted with diethyl ether (2 × 30 mL). The combined organic phases were dried over anhydrous sodium sulfate. Further purification can be achieved by silica gel column chromatography using hexane/ethyl acetate (v/v, 8 : 1) to obtain the product as colorless viscous oil. Yield was 83%. ^{1}H NMR spectrum (400 MHz, DMSO-d_6): δ 2.314 (d, 1H), δ 2.572 (d, 1H), δ 3.147 (m, 1H), δ 3.461-3.597 (m, 3H), δ 4.199 (d, 2H).

Synthesis of 2-((allyloxy)methyl)thiirane (PEMT).

To a suspension of 2-((allyloxy)methyl)oxirane (6.84 g, 60 mmol) in distilled water (30 mL), was added potassium thiocyanate (23.2 g, 240 mmol) and stirred at 40 °C for 24 h. Afterwards, the organic phase was separated and the aqueous phase was extracted with diethyl ether (2 × 30 mL). The combined organic phases were dried over

anhydrous sodium sulfate. Further purification can be achieved by silica gel column chromatography using hexane/ethyl acetate (v/v, 8 : 1) to obtain the product as colorless viscous oil. Yield was 86%. ^{1}H NMR spectrum (400 MHz, $CDCl_3$): δ 2.314 (d, 1H), δ 2.572 (d, 1H), δ 3.147 (m, 1H), δ 3.461-3.597 (m, 2H), δ 4.05 (d, 2H), δ 5.21 (m, 2H), δ 5.86 (m, 1H).

Synthesis of 5-(4-(diphenylamino)phenyl)thiophene-2-carbaldehyde (TTPA).

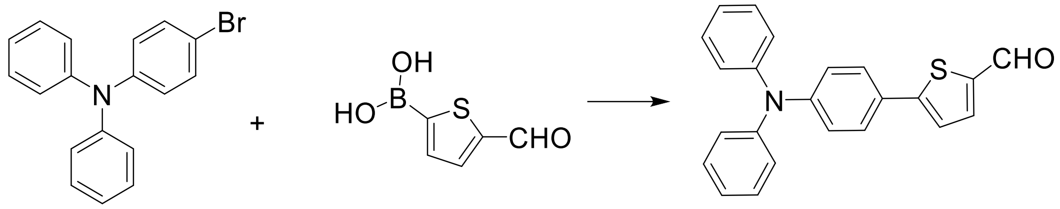

4-Bromotriphenylamine (4.8 g, 15 mmol), 5-formyl-2-thiopheneboronic acid (3.1 g, 20 mmol) and potassium carbonate (4.1 g, 30 mmol) were dissolved in 120 mL anhydrous THF. After stirring at room temperature for 30 min, tetrakis(triphenylphosphine)palladium (200 mg) was added. The solution was stirred and refluxed at 100 °C for 24 h. After cooling to room temperature, the solvent was removed by evaporation and the residue was dissolved in 150 mL CH_2Cl_2. The organic portion was washed with water and dried with anhydrous Na_2SO_4. Further purification was operated by column chromatography using hexane/ethyl acetate (v/v, 12 : 1) to obtain the product as light yellow solid. Yield was 28%. ^{1}H NMR spectrum (400 MHz, DMSO-d_6): δ 3.527 (s, 12H), δ 3.608 (t, 4H), δ 4.215 (t, 4H), δ 4.434 (s, 4H), δ 4.682 (s, 4H).

Synthesis of bifunctional rhodanine monomer.

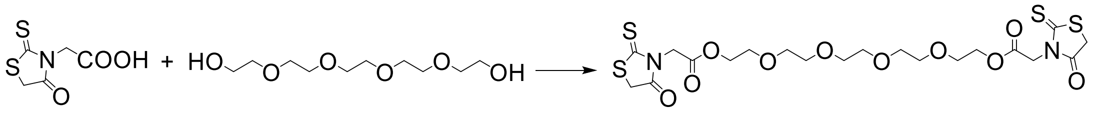

Pentaethylene glycol (4.76 g, 20 mmol) and rhodanine-3-acetic acid (7.6 g, 40 mmol) were added into one 50 mL flask. The mixture was stirred at 160 °C for 6 h. Afterwards, the mixture was diluted with 5 mL ethyl acetate. The combined solution was put into silica gel column chromatography using hexane/ethyl acetate (v/v, 1 : 7) to obtain the product as yellow viscous oil. Yield was 33%. ^{1}H NMR spectrum (400 MHz, DMSO-d_6): δ 3.527 (s, 12H), δ 3.608 (t, 4H), δ 4.215 (t, 4H), δ 4.434 (s, 4H), δ 4.682 (s, 4H).

Synthesis of rhodanine-containing methyl methacrylate (MRDA).

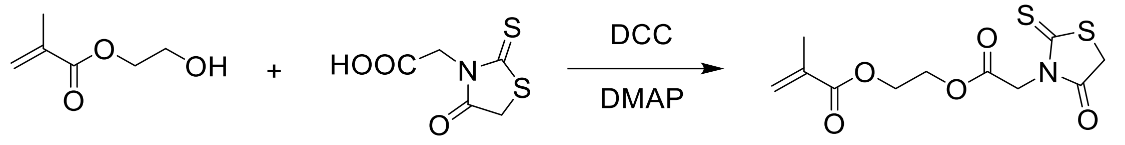

2-Hydroxyethyl methacrylate (6.5 g, 50 mmol), rhodanine-3-acetic acid (4.78 g, 25 mmol) and 4-dimethylaminopyridine (DMAP, 480 mg, 4 mmol) were dissolved in 100 mL anhydrous CH_2Cl_2. Then dicyclohexylcarbodiimide (DCC, 6.7 g, 33 mmol) in 20 mL anhydrous CH_2Cl_2 was added dropwise at 0°C. After stirring at room temperature for 24 h and filtration, the reaction solution washed with H_2O three times. Then the solution was concentrated by evaporator and put into silica gel column chromatography using hexane/ethyl acetate (v/v, 5 : 2) to obtain the product as orange viscous liquid. Yield was 35%. ^{1}H NMR spectrum (400 MHz, DMSO-d_6): δ 1.879 (s, 3H), δ 4.289 (m, 2H), δ 4.366 (m, 2H), δ 4.426 (s, 2H), δ 4.682 (s, 2H), δ 5.702 (s, 1H), δ 6.039 (s, 1H).

Model reaction of rhodanine-aldehyde condensation

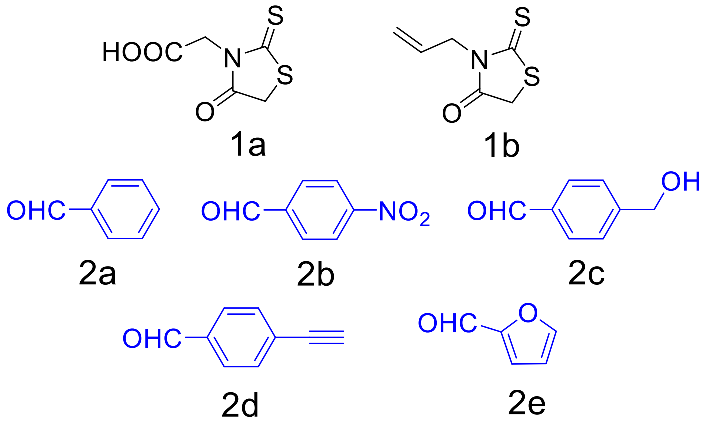

A typical procedure is as follow: 1 (0.2 mmol), 2 (0.2 mmol) and TEA (0.1 mmol) were dissolved in 0.5 mL DMSO-d6 and transferred into a NMR tube. Then the tube was sealed and immersed in an oil bath at 70 °C. After the certain reaction time, the conversions were analyzed by NMR measurement.

Ring-opening polymerization of thiiranes using rhodanine and derivatives as initiators

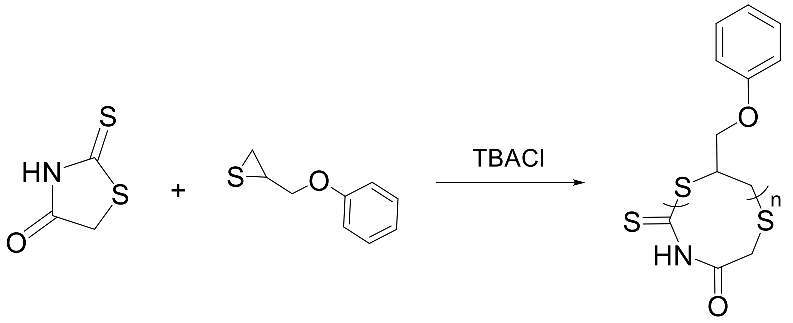

A typical procedure is as follow: rhodanine (33 mg, 0.25 mmol), POMT (830 mg, 5 mmol) and tetrabutylammonium chloride (41 mg, 0.15 mmol) were dissolved in NMP to obtain 2.5 mL solution and transferred into a transparent glass tube. After two freeze-pump-thaw cycles, the tube was sealed and immersed in an oil bath at 75 °C. After 24 h reaction, the solution was precipitated into methanol several times and the product as yellow viscous solid was obtained after dried in vacuum.

Synthesis of cyclic brush-like polymer

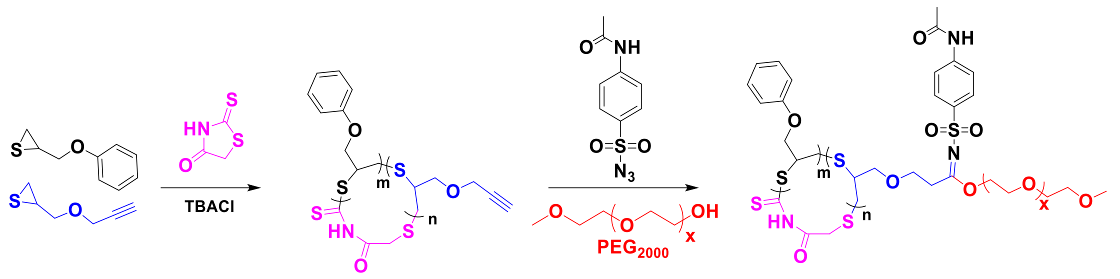

Cyclic-(PPOMT$_{198}$-*r*-PPYMT$_{198}$) (295 mg, 1 mmol of alkynyl group), PEG$_{2000}$-OH (6 g, 3 mmol), CuCl (30 mg, 0.3 mmol) and triethylamine (200 mg, 2 mmol) were dissolved in 15 mL CH_2Cl_2. Then 4-acetamidobenzenesulfonyl azide (720 mg, 3 mmol) in 5 mL CH_2Cl_2 was slowly added into the reaction mixture under an argon atmosphere. After the reaction had been carried out at room temperature for 12 h, the mixture was precipitated into diethyl ether three times. Then the crude product was dissolved in methanol and further purified by dialysis (cut off 3500 Da MWCO). The methanol solvent was changed about 8 h later, and it was changed for at least five times at 8 h periods. The resulting cyclic brush-like polymer was obtained as milk-white solid after concentration.

Synthesis of multicyclic polymers with cyclic units in the backbone.

Synthesis of the precursor polymer: 1d (292 mg, 0.5 mmol), terephthalaldehyde 3f (67 mg, 0.5 mmol) and trimethylamine (25 mg, 0.25 mmol) were dissolved in DMF to obtain 2.5 mL solution and immersed in an oil bath at 70 °C. After 3 h reaction, the solution precipitated into methanol several times and the product precursor polymer as light brown solid was obtained after dried in vacuum.

The precursor polymer (72.2 mg, 0.2 mmol heterocycle), POMT (664 mg, 4 mmol) and TBACl (56 mg, 0.2 mmol) were dissolved in NMP to obtain 2 mL solution and transferred into a transparent glass tube. After two freeze-pump-thaw cycles, the tube was sealed and immersed in an oil bath at 75 °C. After 18 h reaction, the solution was precipitated into methanol several times and the product as light brown solid was

obtained after dried in vacuum.

Synthesis of red/near-infrared AIE multicyclic polymer with pendant cyclic units.

2-[[[(2-Carboxyethyl)thio]thioxomethyl]thio]-2-methylpropanoic acid (26.8 mg, 0.1 mmol), MMA (1200 mg, 12 mmol), MRDA (303 mg, 1 mmol) and AIBN (3.2 mg, 0.02 mmol) were dissolved in DMF to obtain 10 mL solution and transferred into a transparent glass tube. After three freeze-pump-thaw cycles, the tube was sealed and immersed in an oil bath at 65 °C. After 12 h reaction, the solution was precipitated into methanol several times and the copolymer 1 as light yellow powder was obtained after dried in vacuum.

Then this copolymer 1 and 40 fold excess AIBN were dissolved in 50 mL THF to remove RAFT end groups. After three freeze-pump-thaw cycles, the solution was sealed and immersed in an oil bath at 80 °C. After 16 h reaction, the solution was precipitated into diethyl ether several times and the copolymer 2 as white powder was obtained after dried in vacuum.

Copolymer 2 without RAFT end groups (300 mg, 0.2 mmol RDA units), 5-(4-(diphenylamino)phenyl)thiophene-2-carbaldehyde (74.6 mg, 0.21 mmol) and TEA (10 mg, 0.1 mmol) were dissolved in DMF to obtain 3 mL solution and immersed in an oil bath at 70 °C. After 1 h reaction, the solution was precipitated into diethyl ether and hexane (1 : 1) several times and the red/near-infrared AIE copolymer 3 as dark red powder was obtained after dried in vacuum.

Copolymer 3 (57 mg, 0.03 mmol RDA units), POMT (150 mg, 0.9 mmol) and tetrabutylammonium chloride (13 mg, 0.045 mmol) were dissolved in NMP to obtain 0.75 mL solution and transferred into a transparent glass tube. After two freeze-pump-thaw cycles, the tube was sealed and immersed in an oil bath at 75 °C. After 10 h reaction, the solution was precipitated into methanol several times and the red/near-infrared AIE multicyclic polymer with pendant cyclic units as red solid was obtained after dried in vacuum.

Supplementary Figure

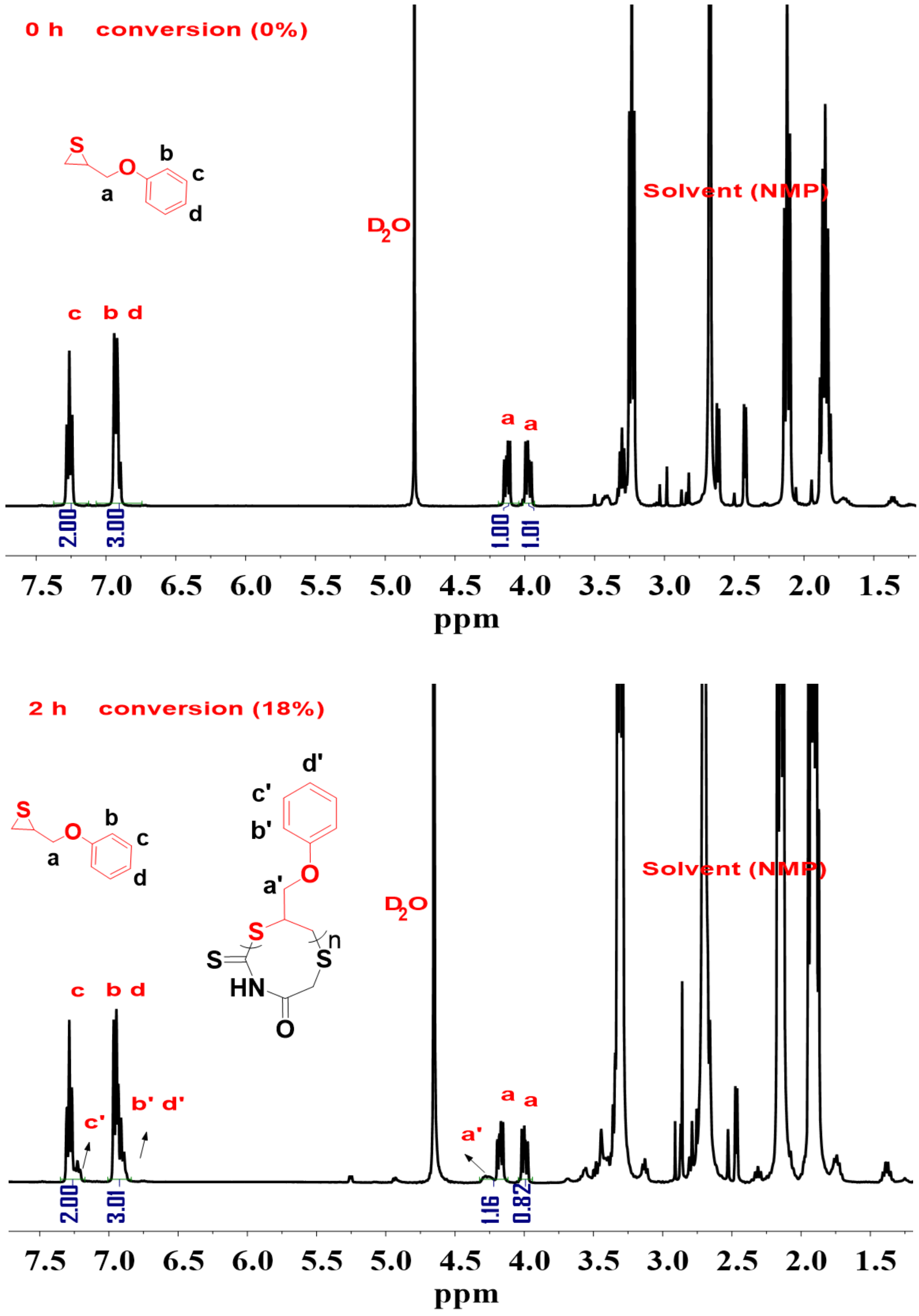

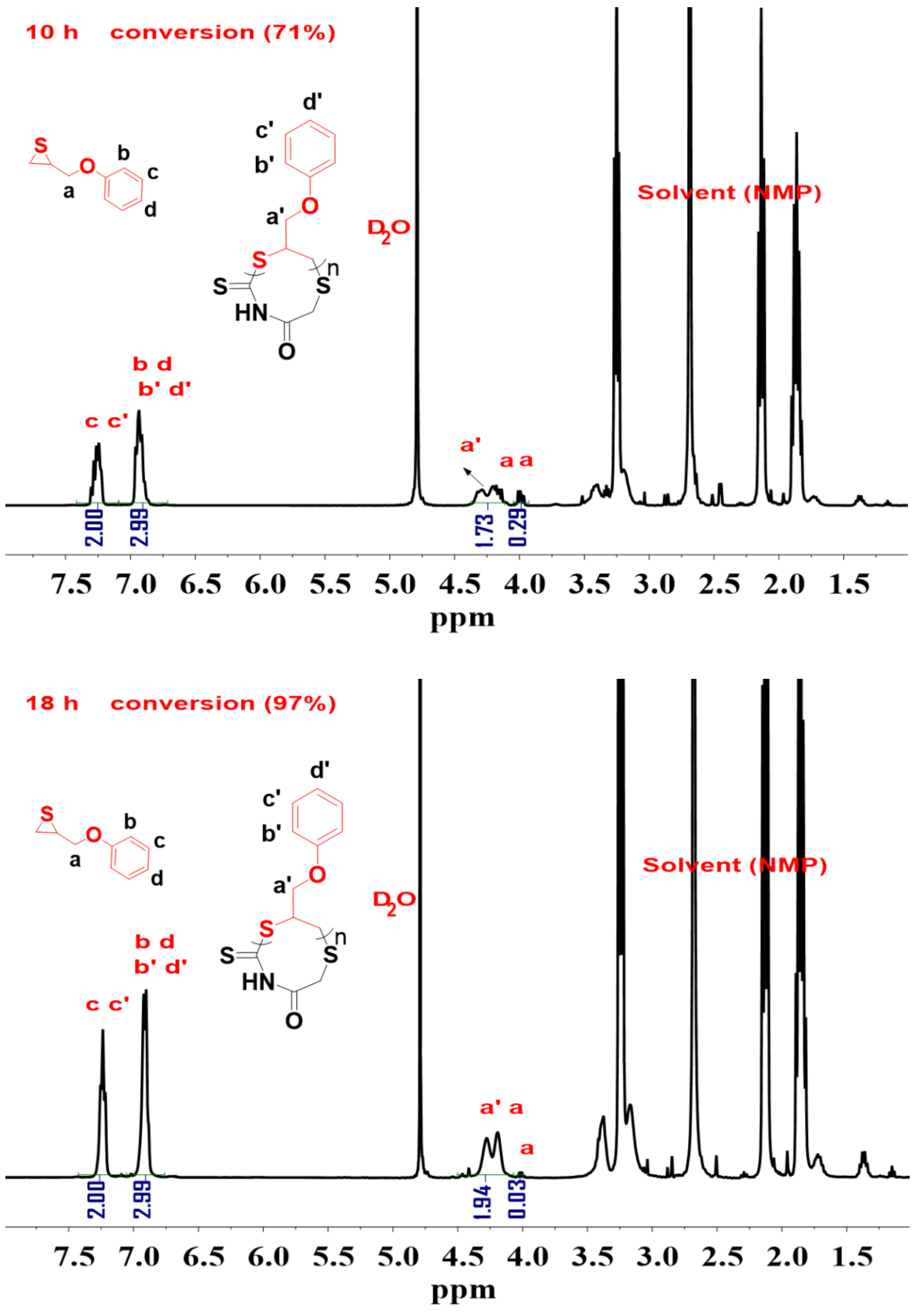

Supplementary Figure 1. ^{1}H NMR trace during the formation of cyclic polymer: Conversion (POMT) = (1-I_a) × 100%; I_a denotes the protons integral values of methylene at unreacted POMT (δ = 4.00 ppm).

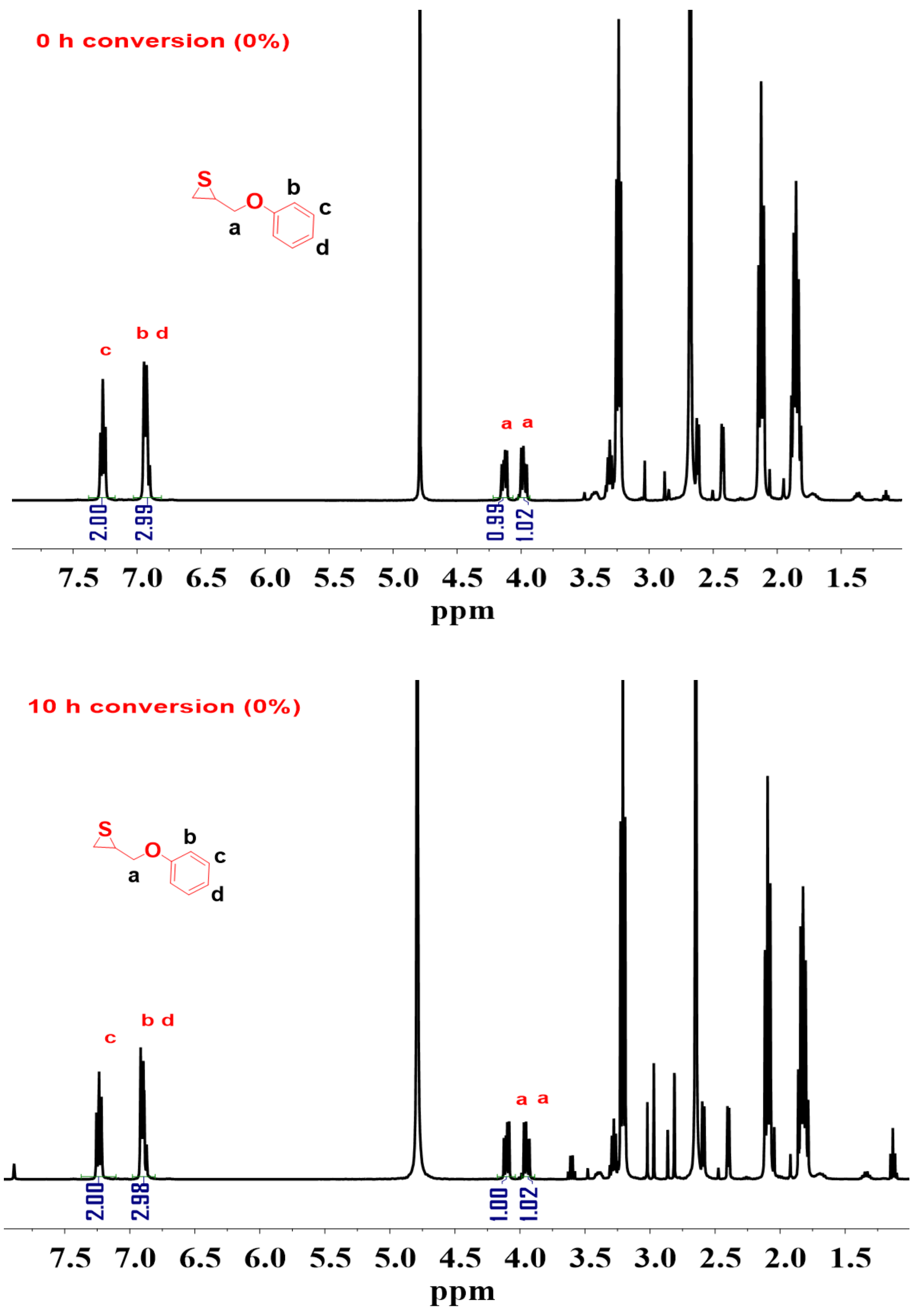

Supplementary Figure 2. ^{1}H NMR trace without adding rhodanine as the initiator

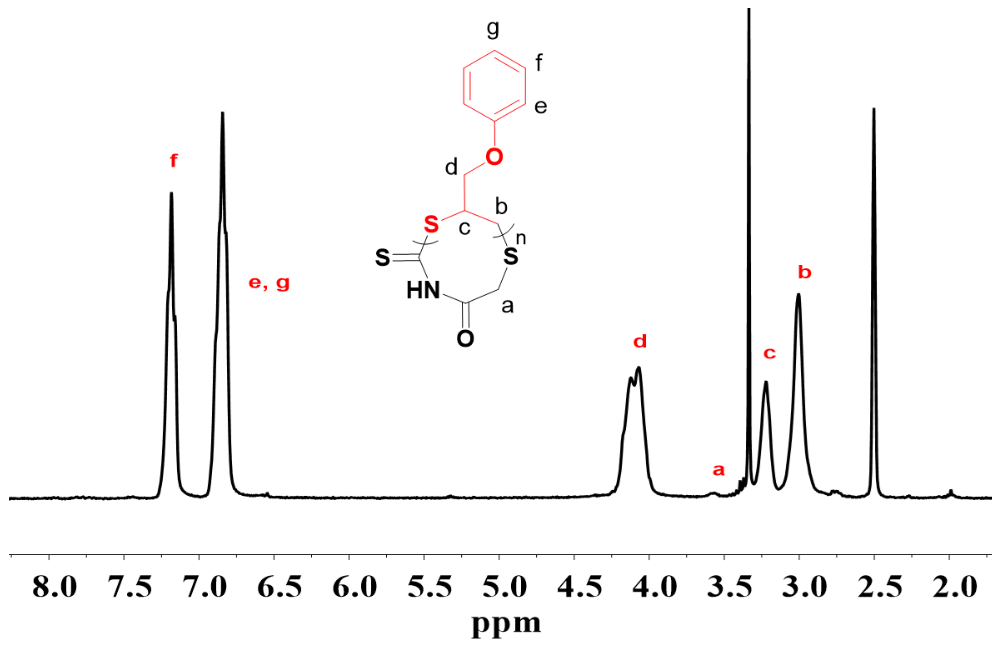

Supplementary Figure 3. The ^{1}H NMR spectrum of the cyclic polymer in entry 1, Table 1

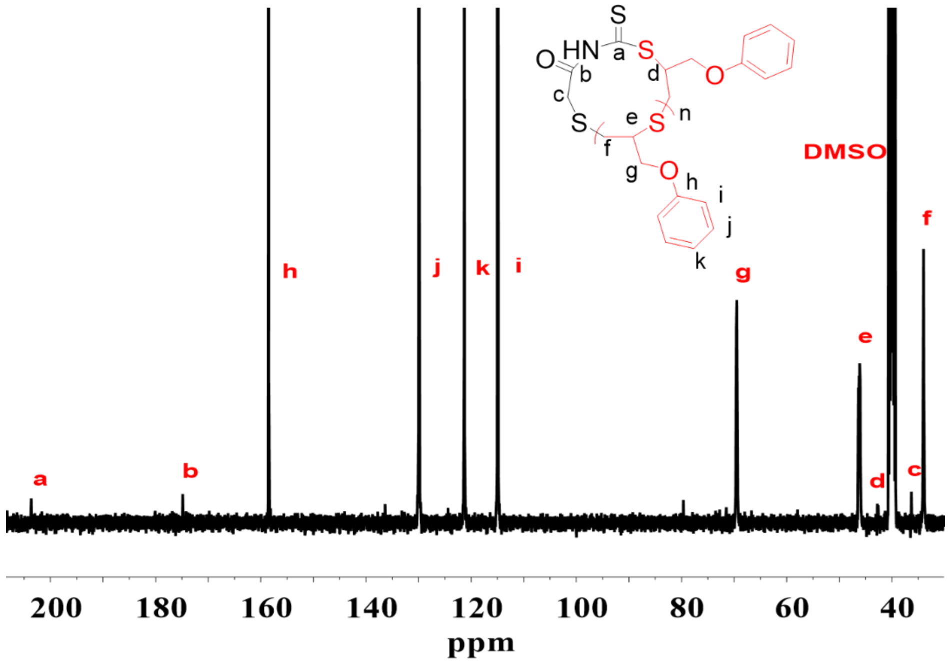

Supplementary Figure 4. ^{13}C NMR spectrum of the cyclic polymer in entry 1, Table1

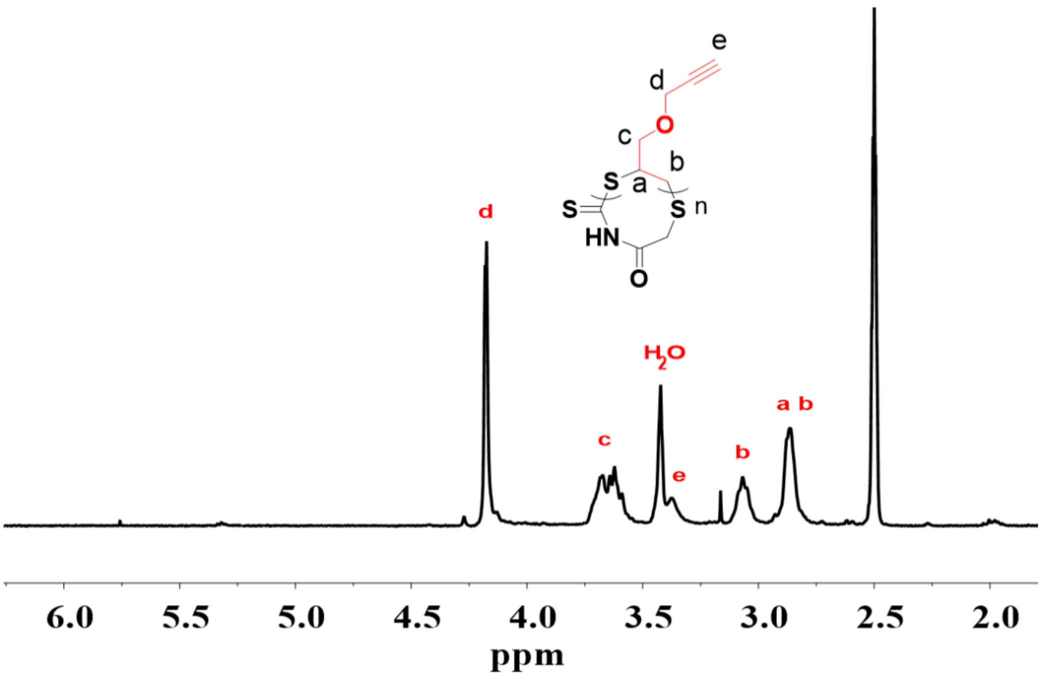

Supplementary Figure 5. The ^{1}H NMR spectrum of the cyclic polymer in entry 2, Table 1

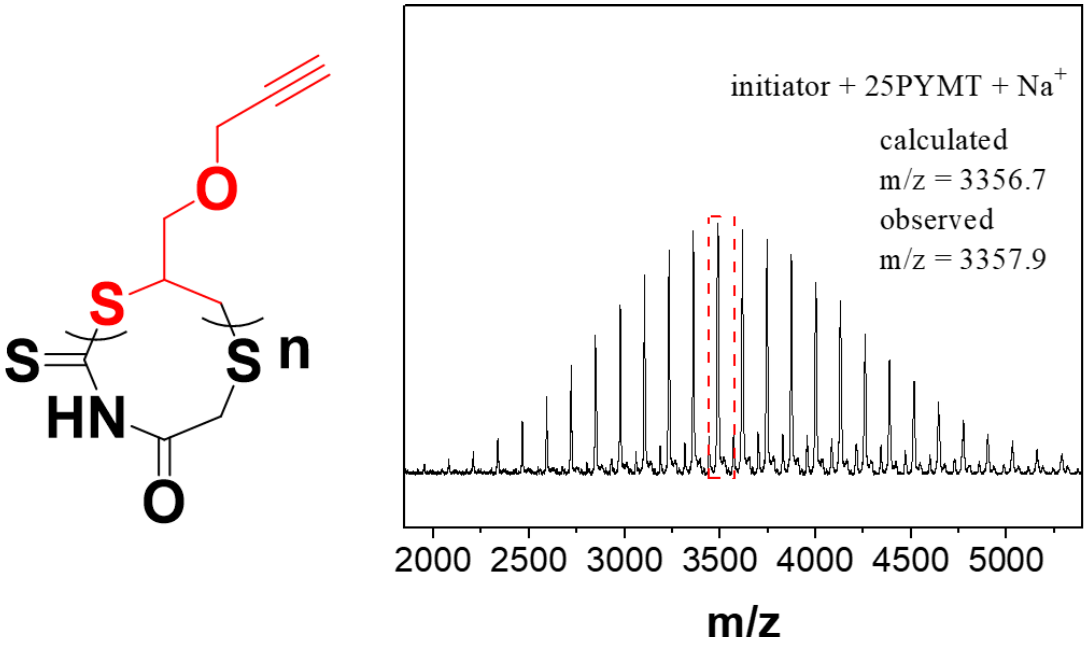

Supplementary Figure 6. The MALDI-TOF MS spectrum of the cyclic PPYMT in entry 2, Table 1

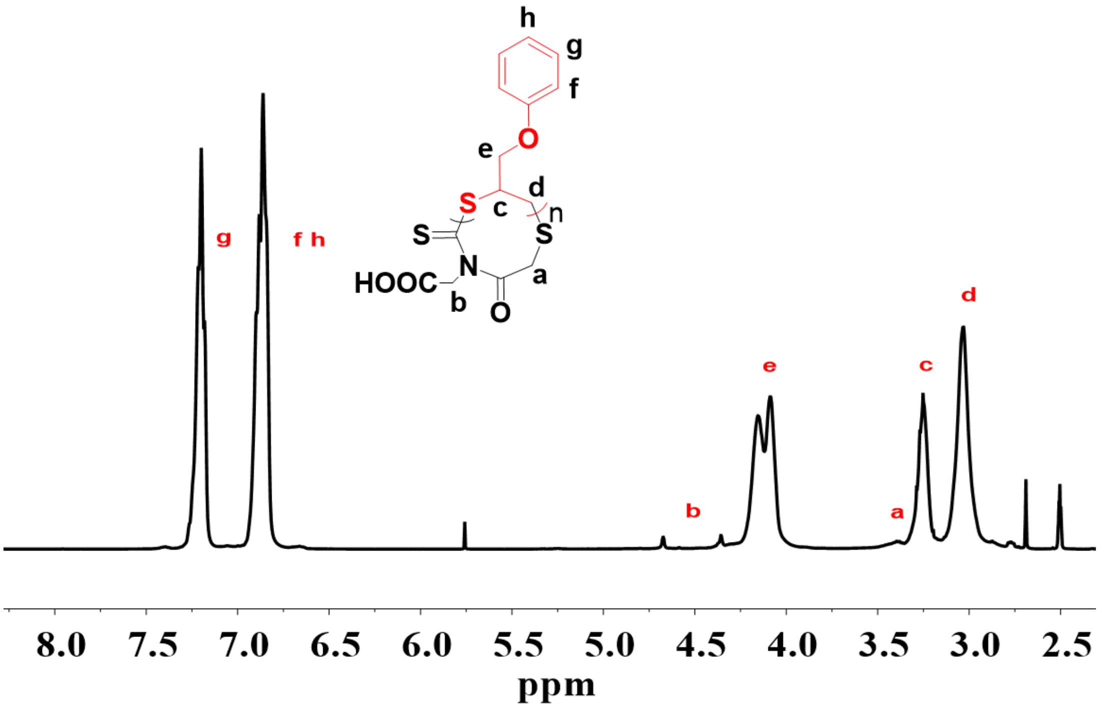

Supplementary Figure 7. The ^{1}H NMR spectrum of the cyclic polymer in entry 3, Table 1 using *N*-substituent rhodanine derivative as the initiator

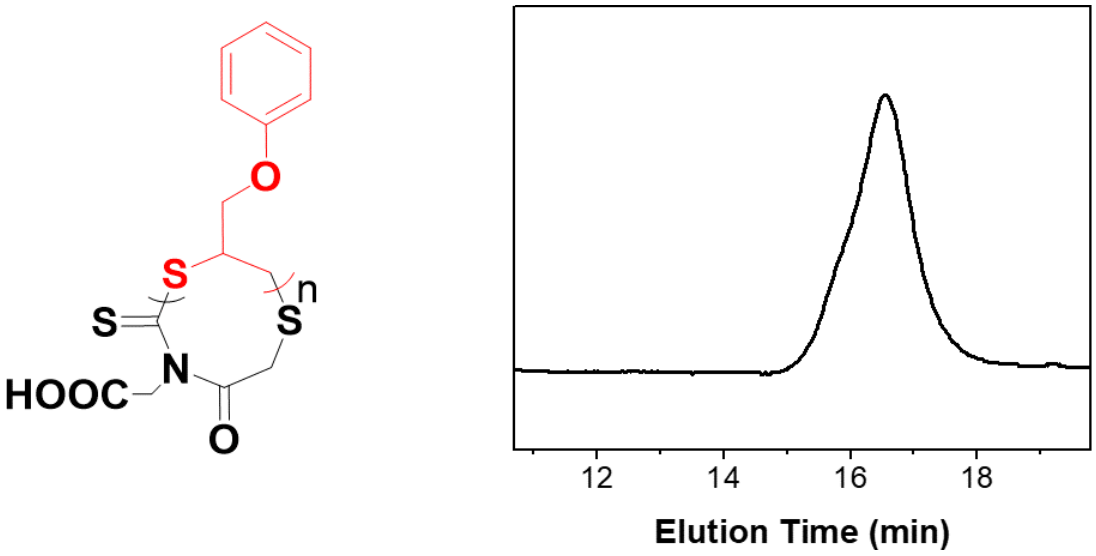

Supplementary Figure 8. SEC curve of resulting polymer in entry 3, Table 1 using *N*-substituent rhodanine derivative as the initiator

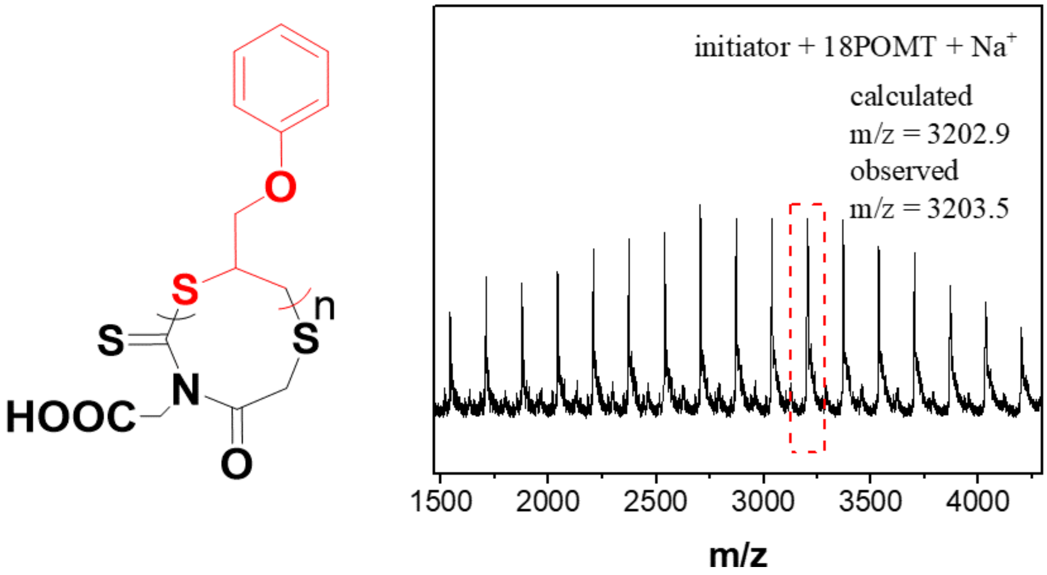

Supplementary Figure 9. MALDI-TOF MS spectrum of resulting polymer in entry 3, Table 1 using *N*-substituent rhodanine derivative as the initiator

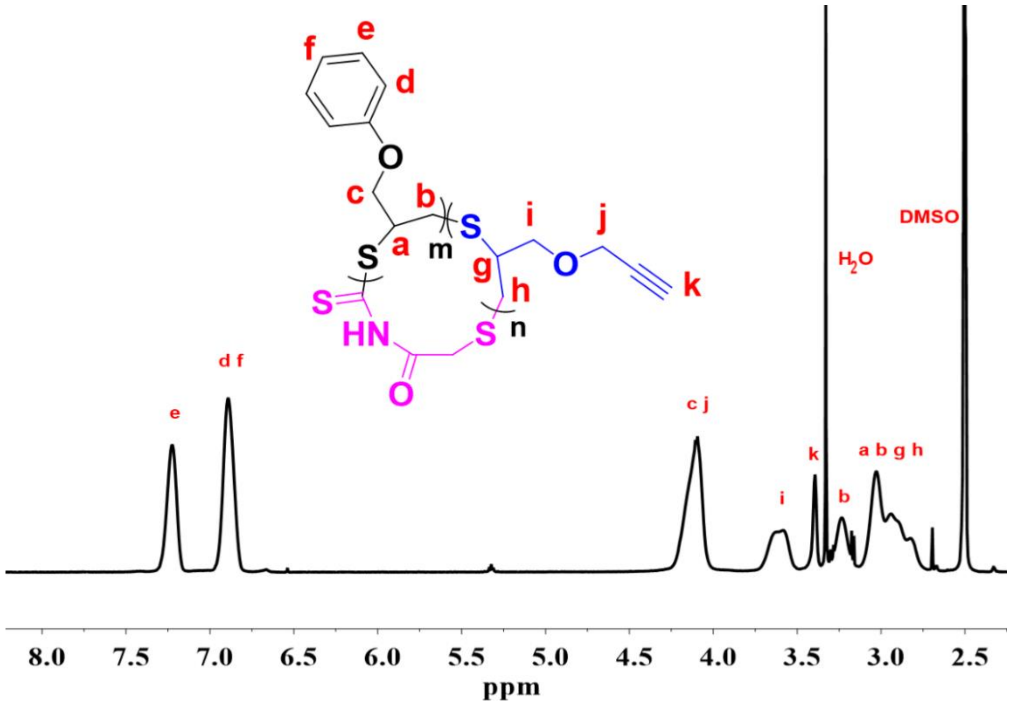

Supplementary Figure 10. The ^{1}H NMR spectrum of cyclic random copolymer in entry 4, Table 1

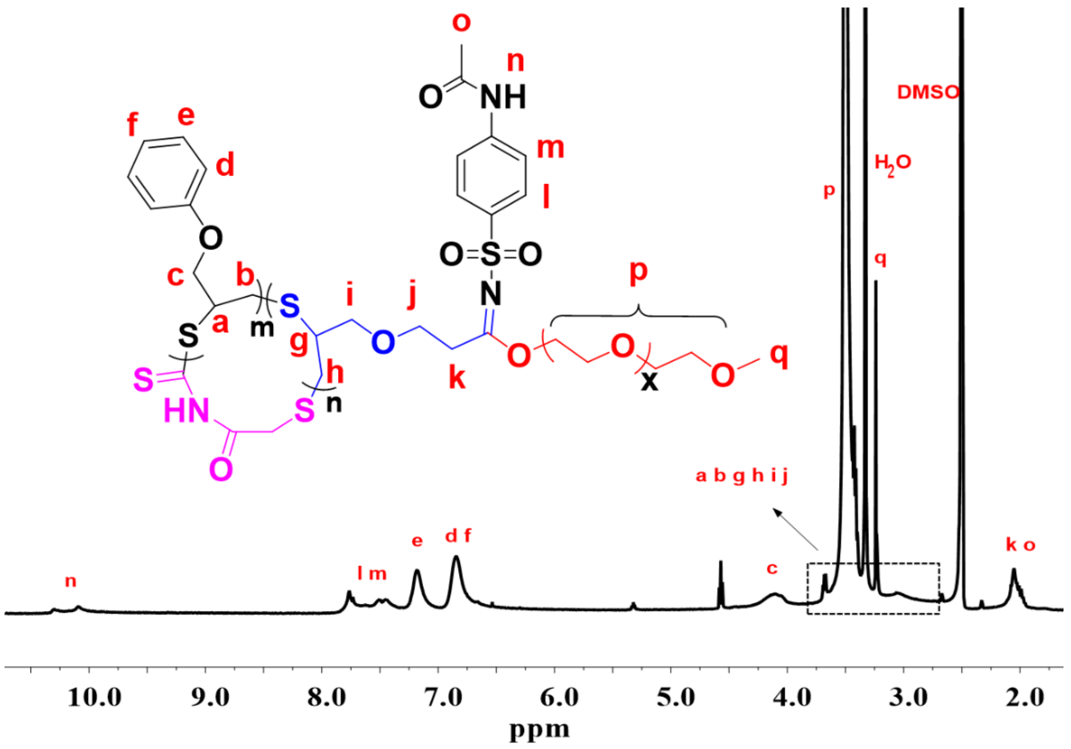

Supplementary Figure 11. The ^{1}H NMR spectrum of the cyclic graft copolymer

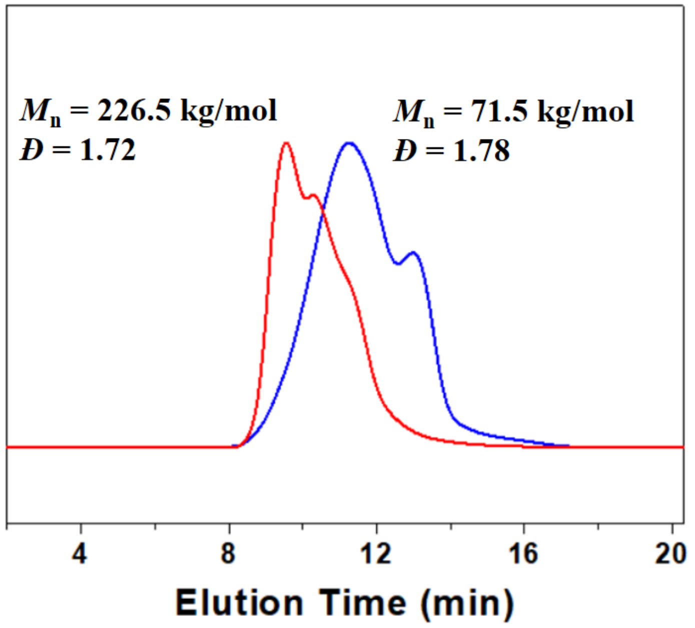

Supplementary Figure 12. The SEC curves of the cyclic copolymer and cyclic graft copolymer

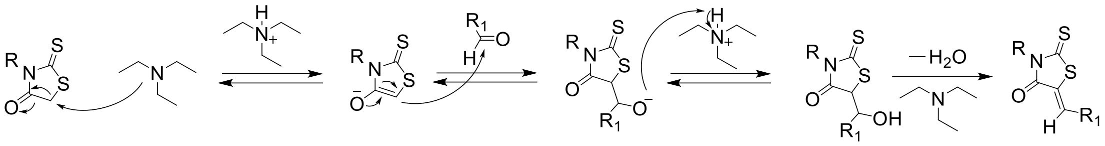

Supplementary Figure 13. The mechanism of RA Knoevenagel reaction

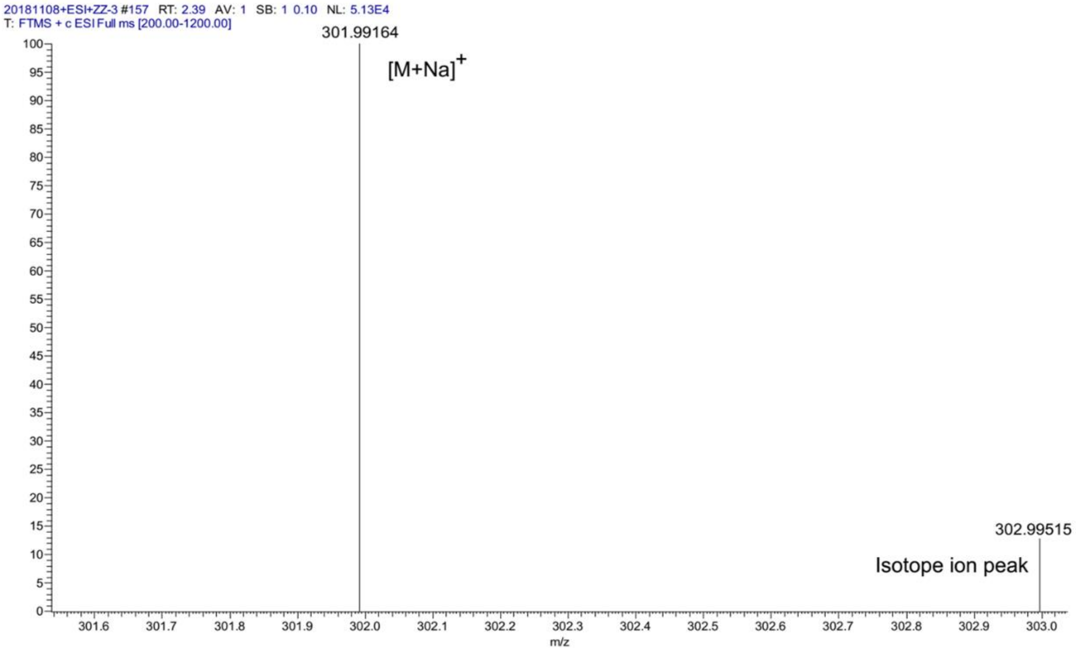

Supplementary Figure 14. The HR-MS analysis of RA reaction in entry 1, Table 2

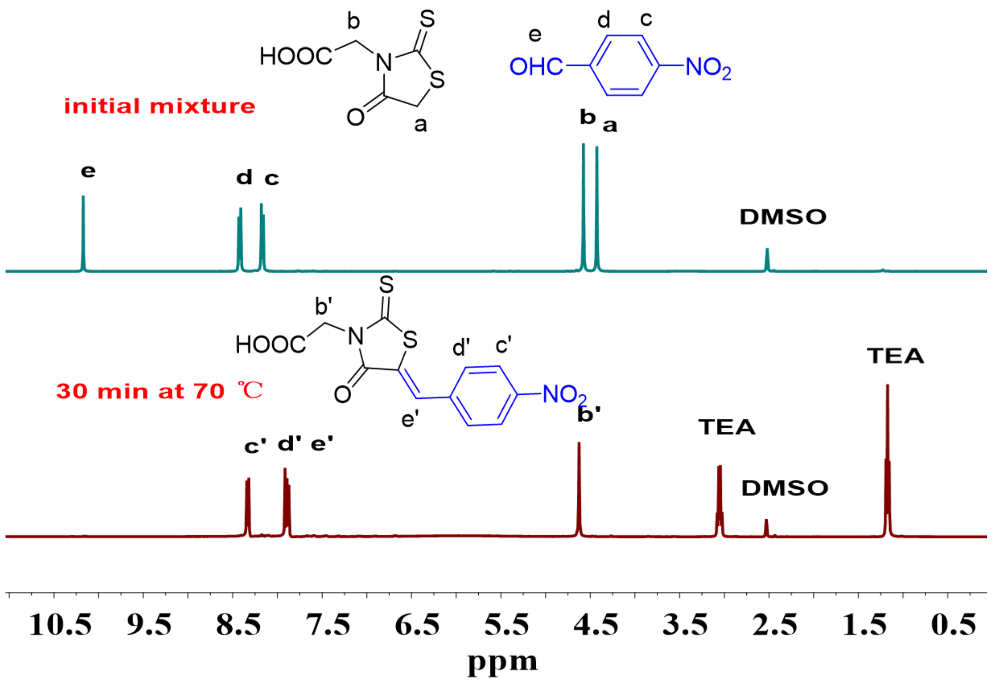

Supplementary Figure 15. The ^{1}H NMR trace of RA reaction in entry 2, Table 2

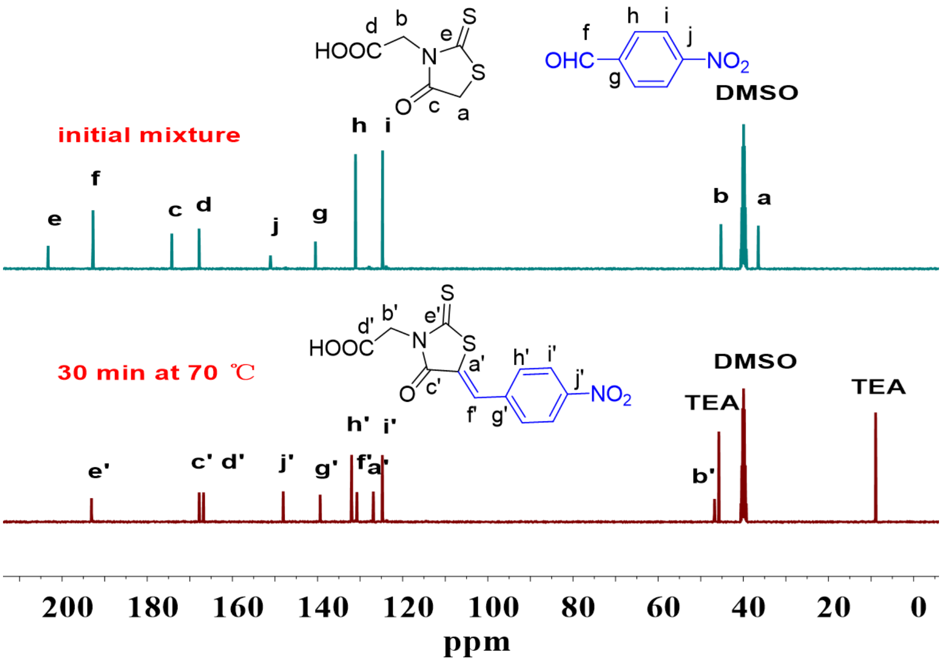

Supplementary Figure 16. The ^{13}C NMR trace of RA reaction in entry 2, Table 2

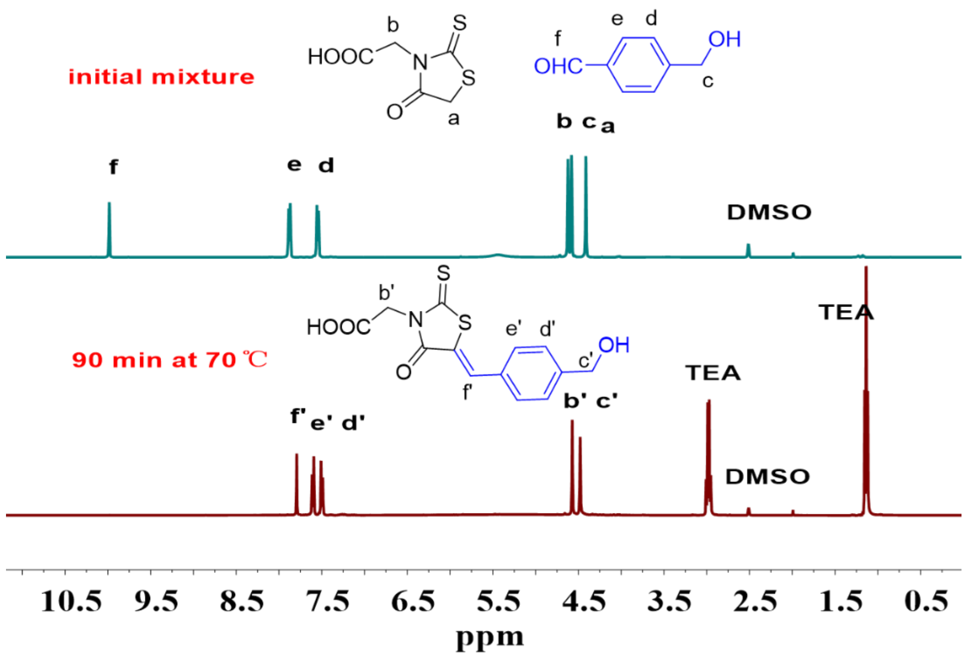

Supplementary Figure 17. The ^{1}H NMR trace of RA reaction in entry 3, Table 2

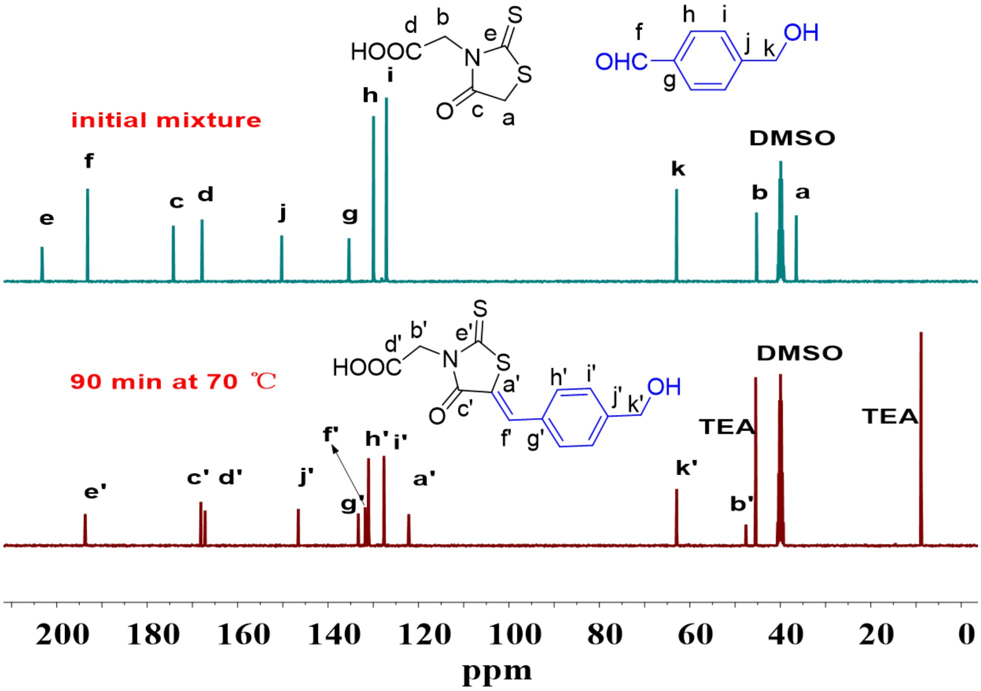

Supplementary Figure 18. The ^{13}C NMR trace of RA reaction in entry 3, Table 2

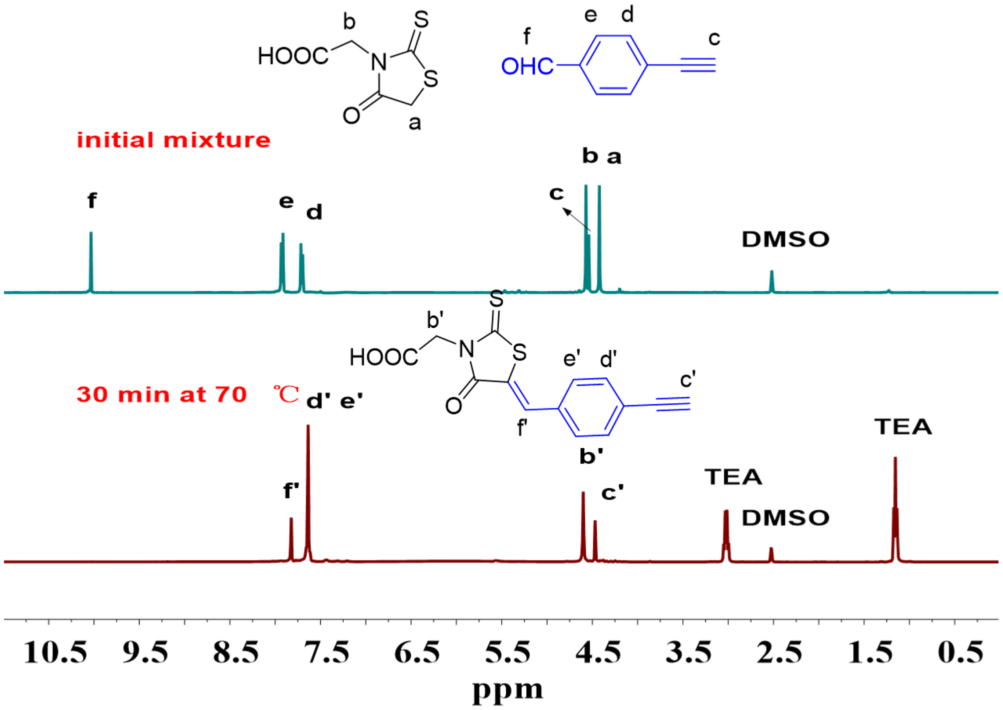

Supplementary Figure 19. The ^{1}H NMR trace of RA reaction in entry 4, Table 2

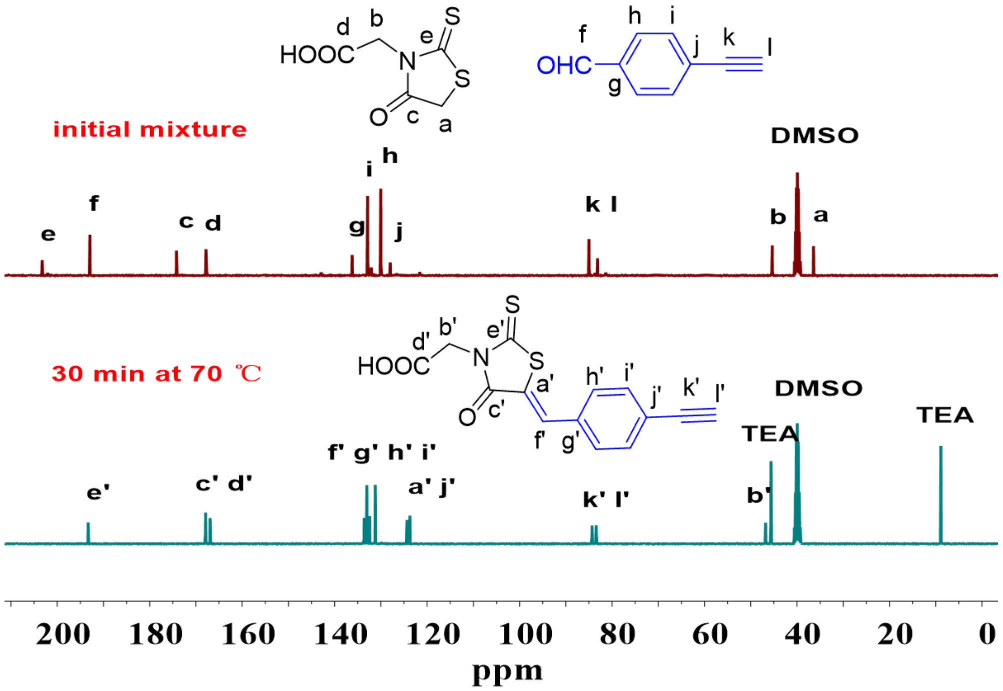

Supplementary Figure 20. The ^{13}C NMR trace of RA reaction in entry 4, Table 2

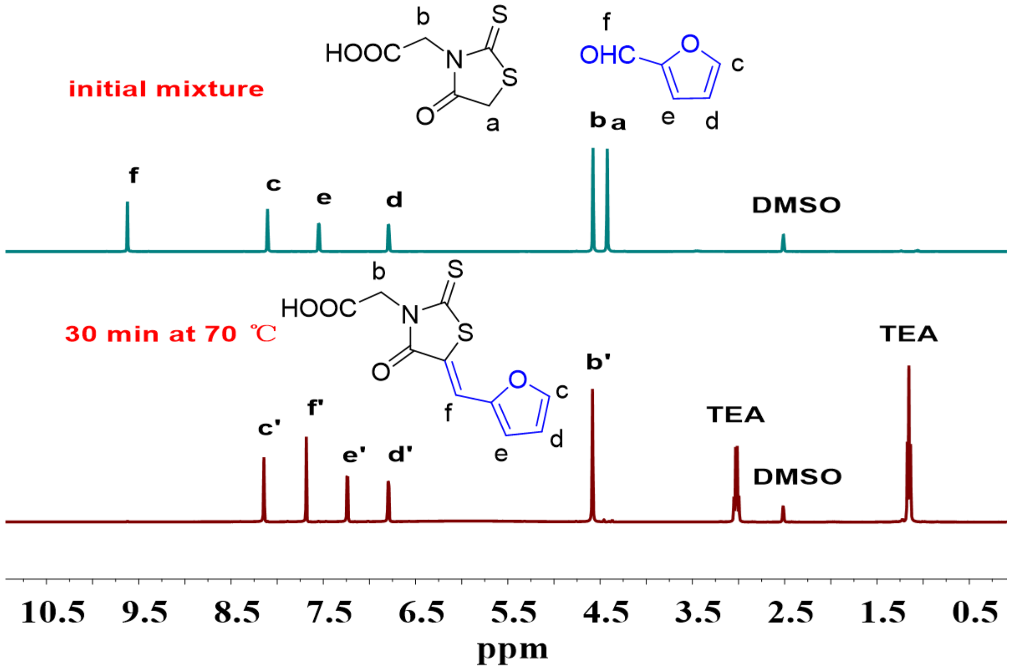

Supplementary Figure 21. The ^{1}H NMR trace of RA reaction in entry 5, Table 2

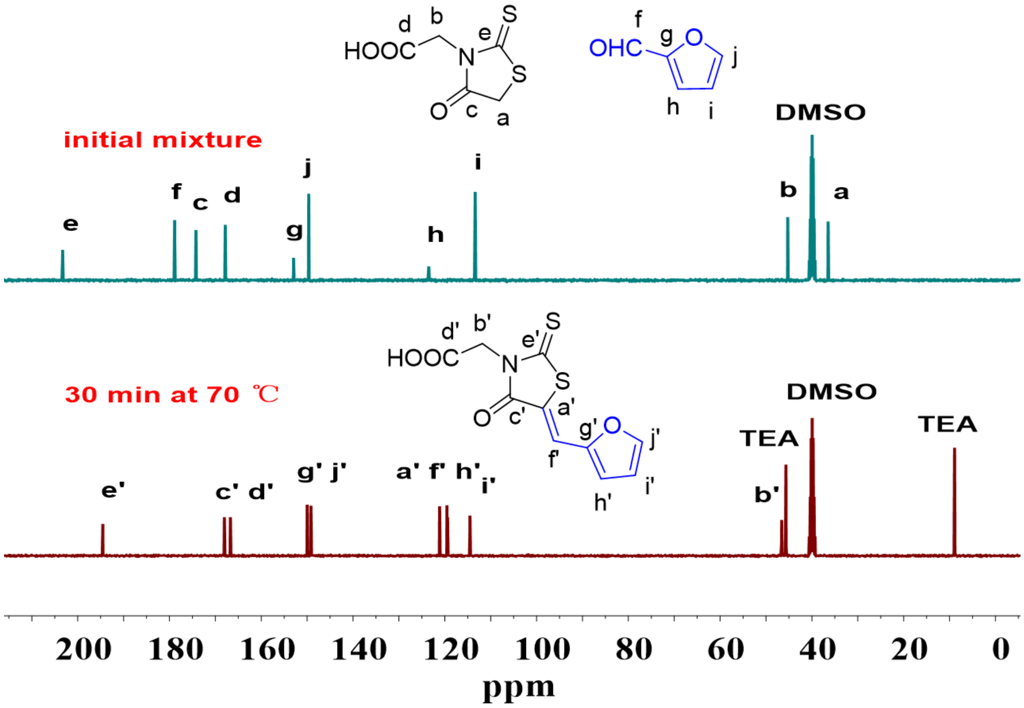

Supplementary Figure 22. The ^{13}C NMR trace of RA reaction in entry 5, Table 2

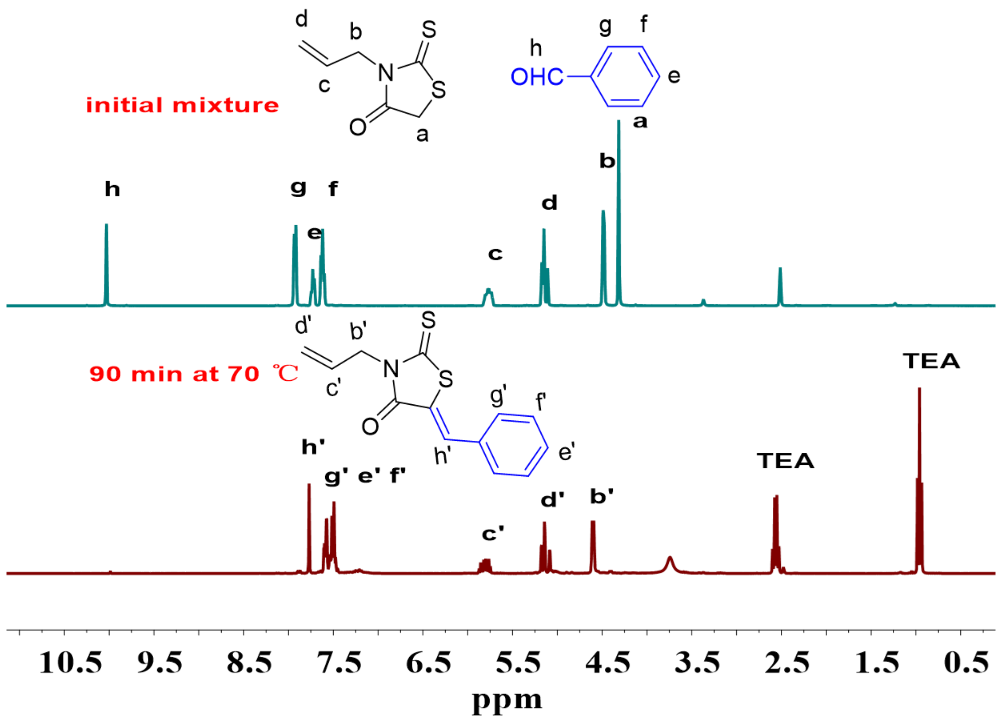

Supplementary Figure 23. The ^{1}H NMR trace of RA reaction in entry 6, Table 2

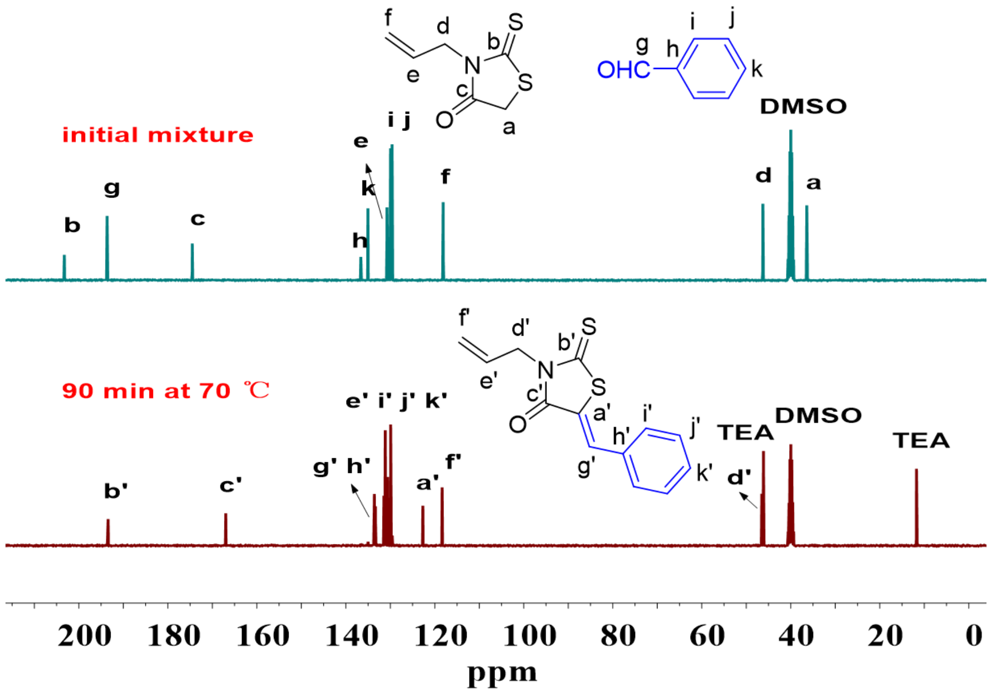

Supplementary Figure 24. The ^{13}C NMR trace of RA reaction in entry 6, Table 2

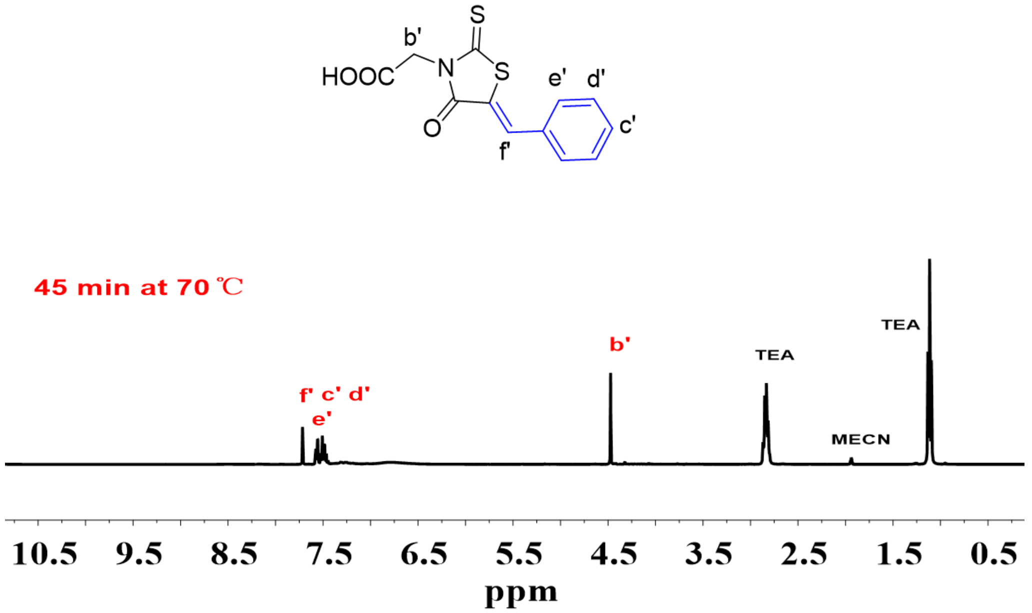

Supplementary Figure 25. The ^{1}H NMR trace of RA reaction in entry 7, Table 2

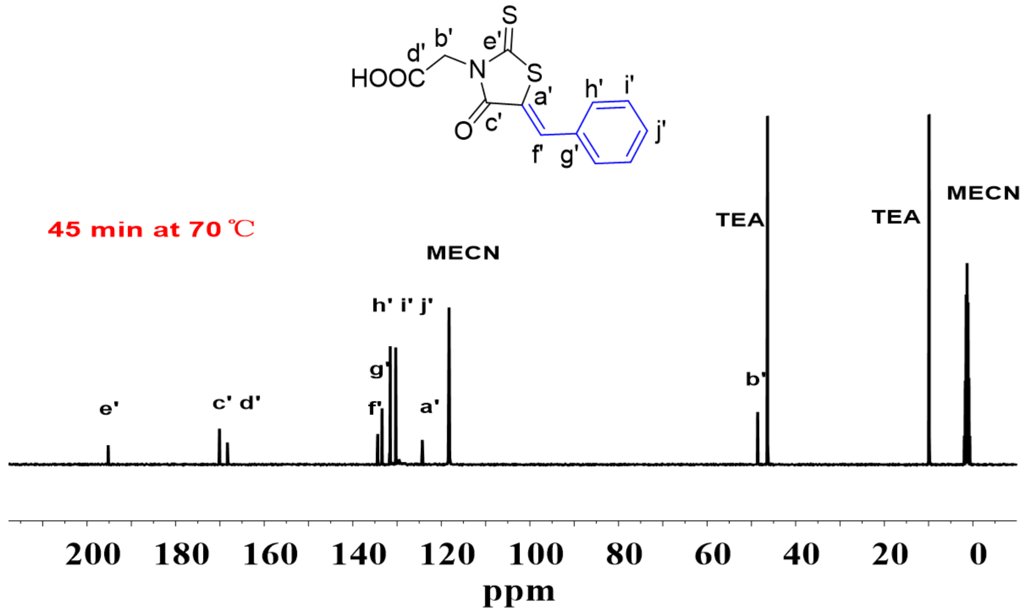

Supplementary Figure 26. The ^{13}C NMR trace of RA reaction in entry 7, Table 2

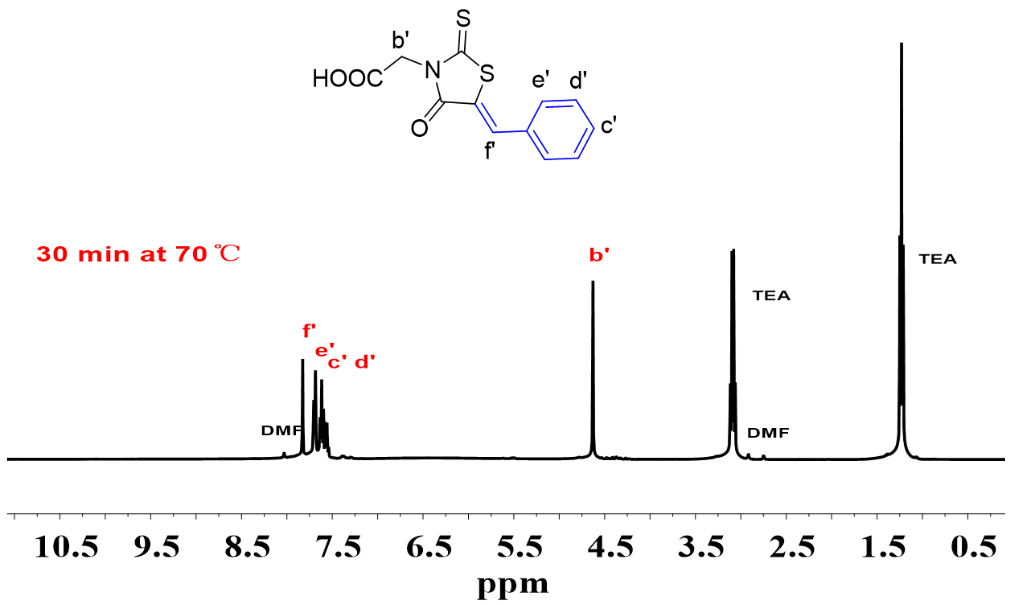

Supplementary Figure 27. The ^{1}H NMR trace of RA reaction in entry 8, Table 2

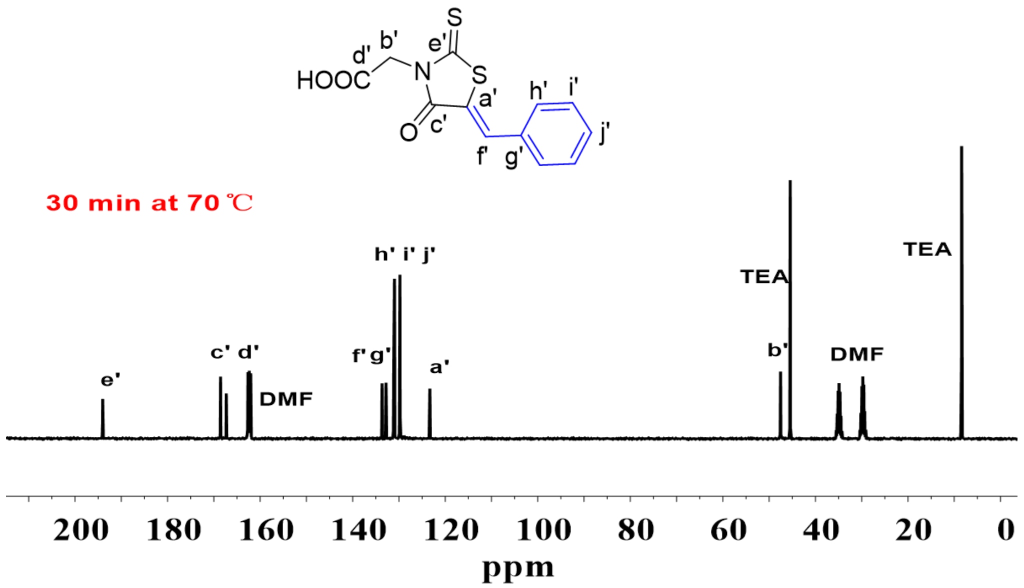

Supplementary Figure 28. The ^{13}C NMR trace of RA reaction in entry 8, Table 2

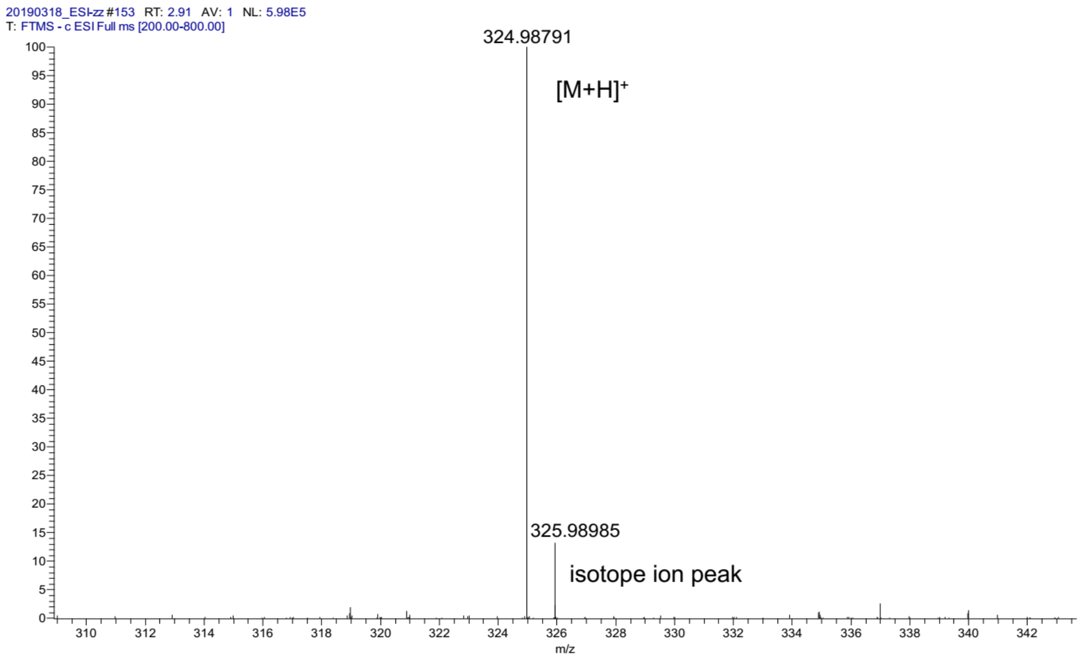

Supplementary Figure 29. The HR-MS analysis of RA reaction in entry 2, Table 2

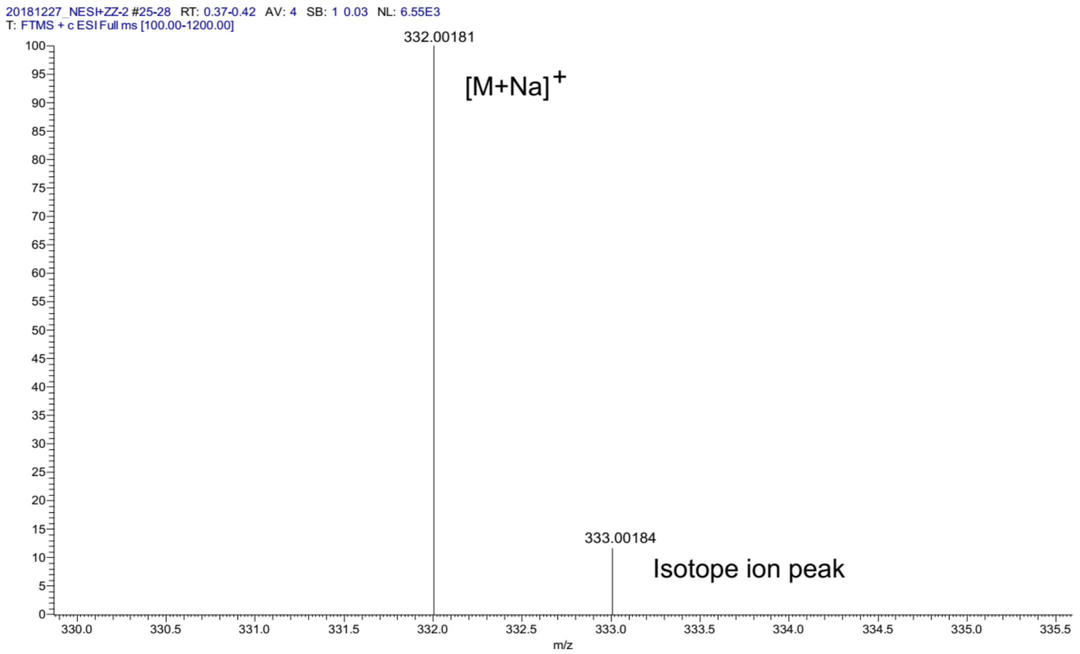

Supplementary Figure 30. The HR-MS analysis of RA reaction in entry 3, Table 2

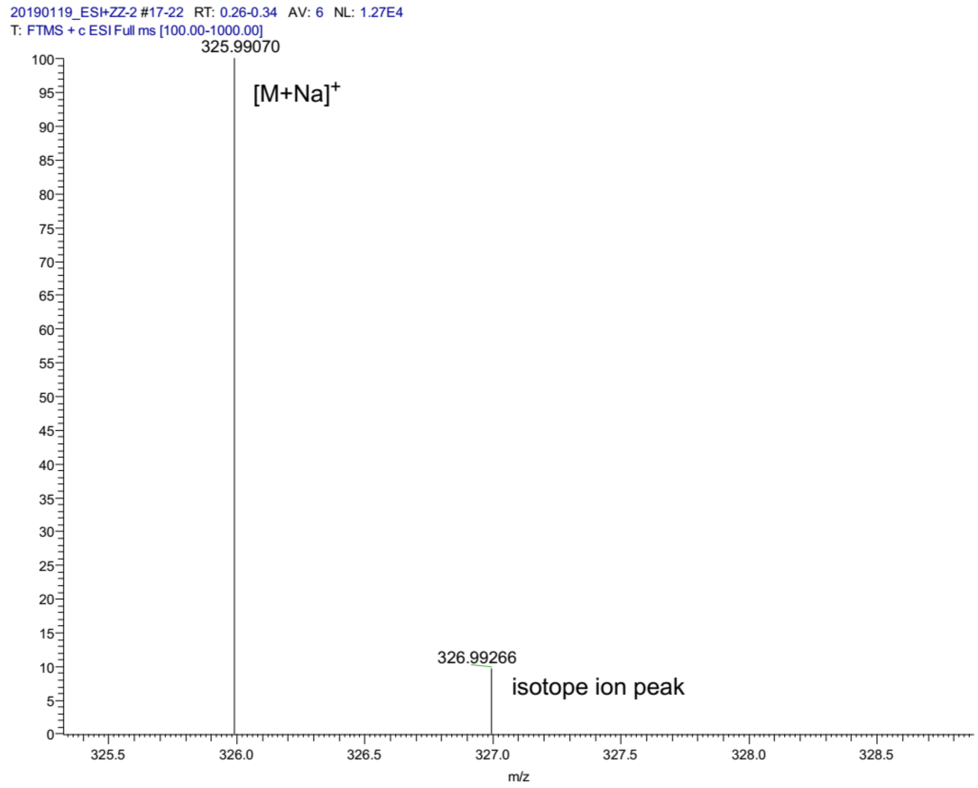

Supplementary Figure 31. The HR-MS analysis of RA reaction in entry 4, Table 2

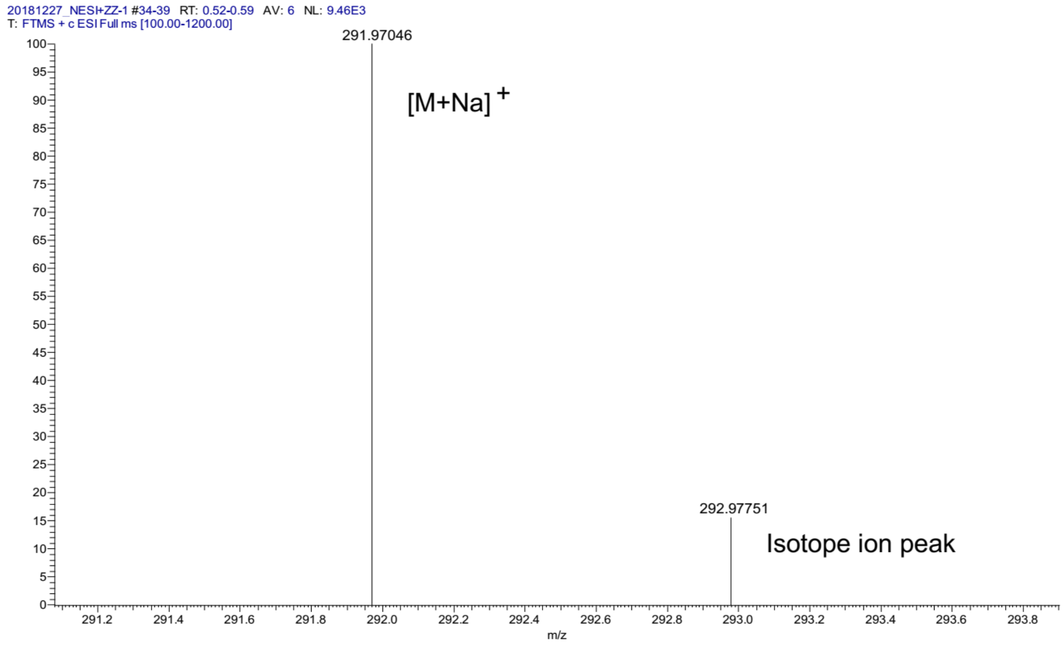

Supplementary Figure 32. The HR-MS analysis of RA reaction in entry 5, Table 2

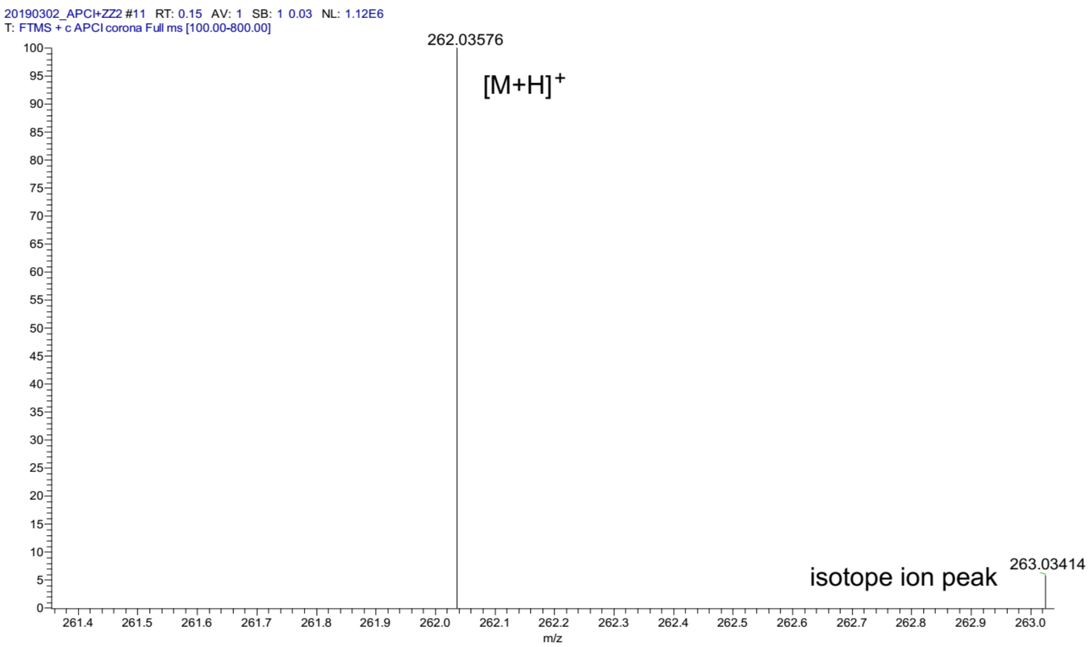

Supplementary Figure 33. The HR-MS analysis of RA reaction in entry 6, Table 2

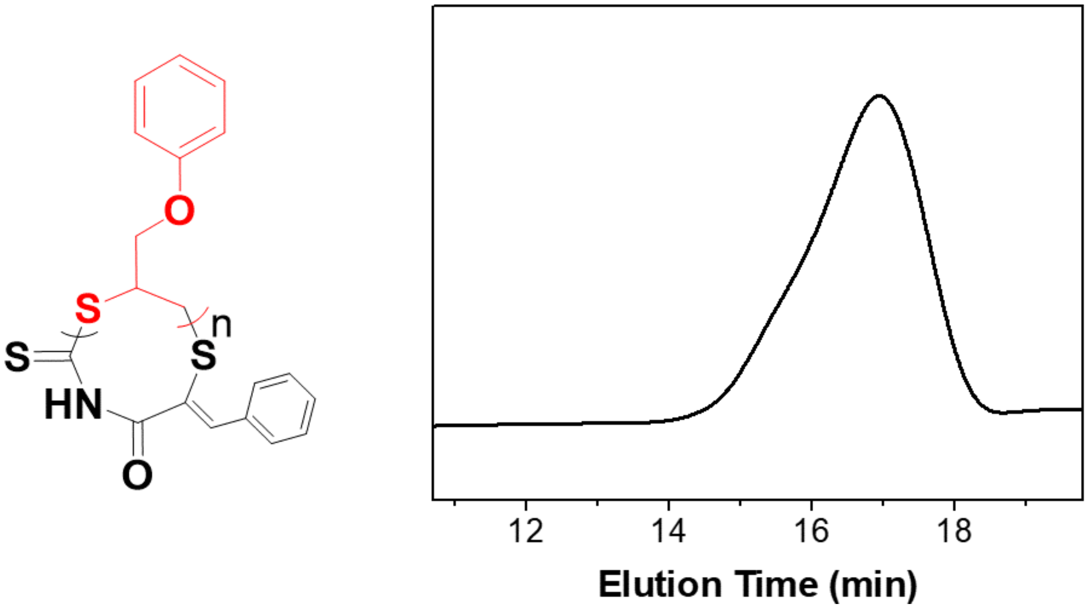

Supplementary Figure 34. SEC curve of resulting polymer using *C*-substituent RA reaction product in Figure 4 as the initiator

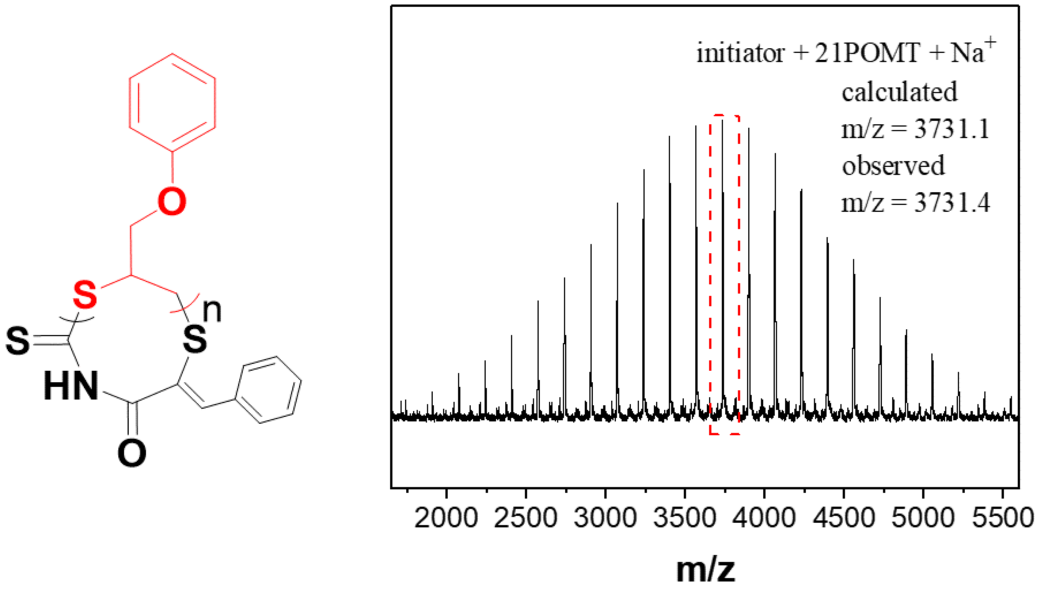

Supplementary Figure 35. MALDI-TOF MS spectrum of resulting polymer using *C*-substituent RA reaction product in Figure 4 as the initiator

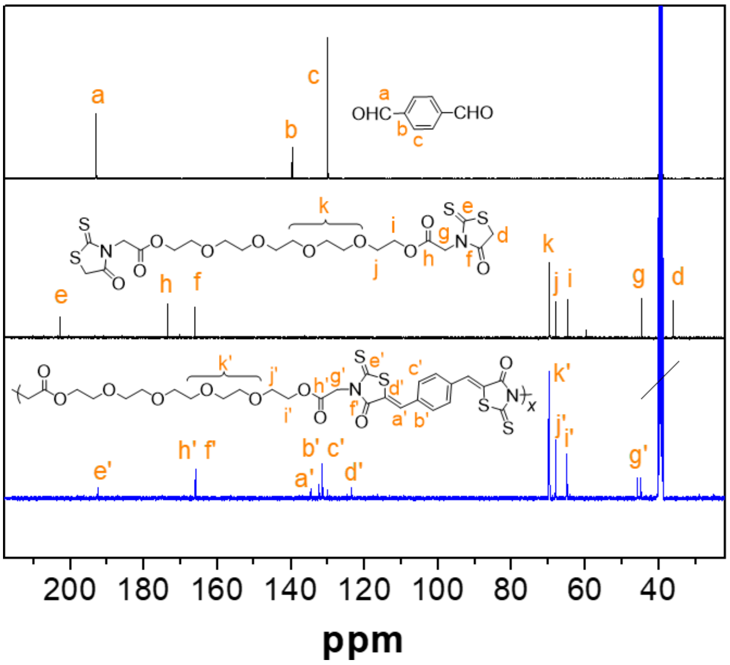

Supplementary Figure 36. ^{13}C NMR spectrum of RA Knoevenagel polymerization product and corresponding monomers

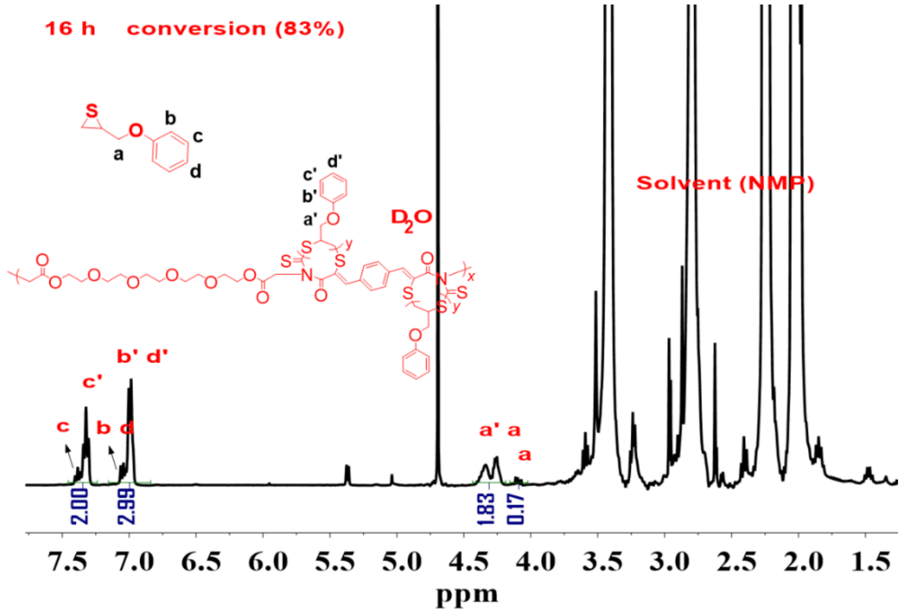

Supplementary Figure 37. ^{1}H NMR spectrum of mixture during the formation of multicyclic copolymer: Conversion (POMT) = (1-I_a) × 100%; I_a denotes the protons integral values of methylene at unreacted POMT (δ = 4.00 ppm)

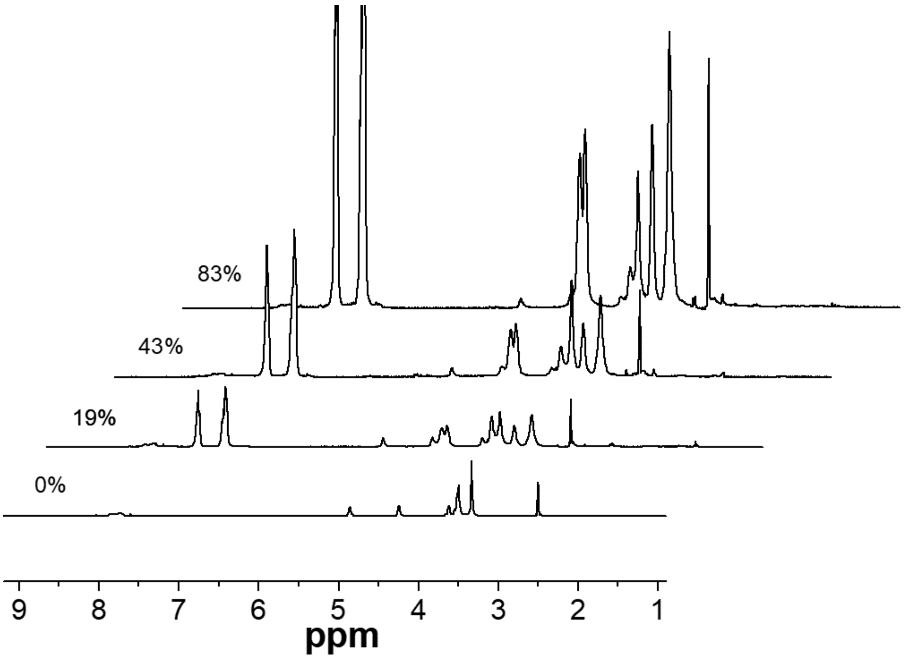

Supplementary Figure 38. The ^{1}H NMR spectra of the multicyclic copolymer with cyclic units in the backbone at different conversions

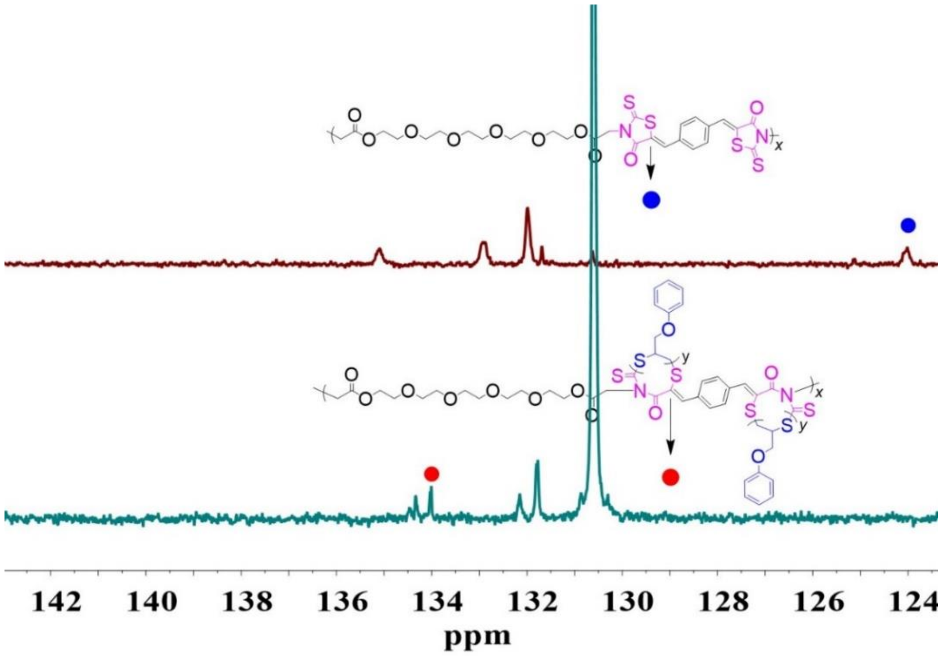

Supplementary Figure 39. ^{13}C NMR analysis of the precursor polymer and multicyclic polymers

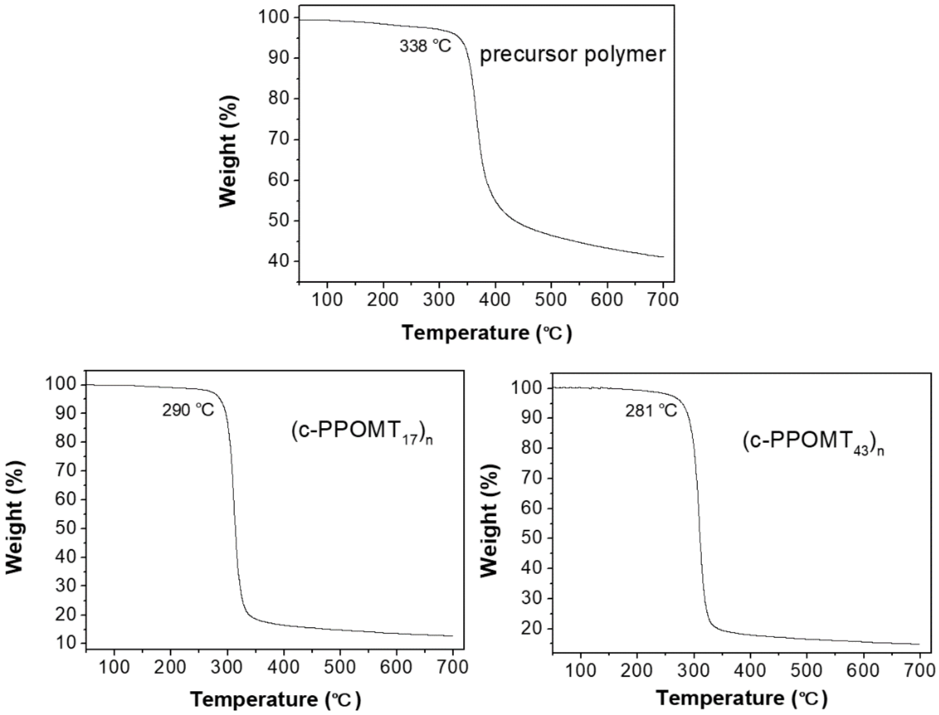

Supplementary Figure 40. TGA of the precursor polymer and multicyclic polymers

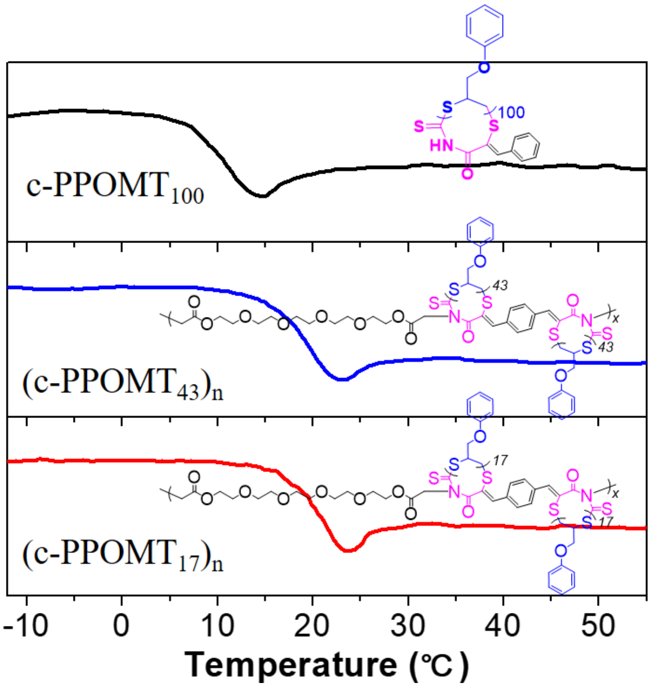

Supplementary Figure 41. DSC curves of multicyclic polymers and single cyclic counterpart

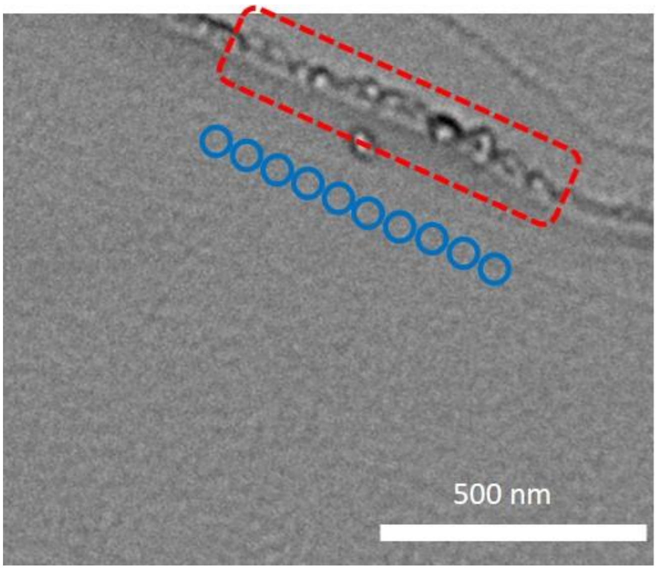

Supplementary Figure 42. TEM image of multicyclic copolymer brush

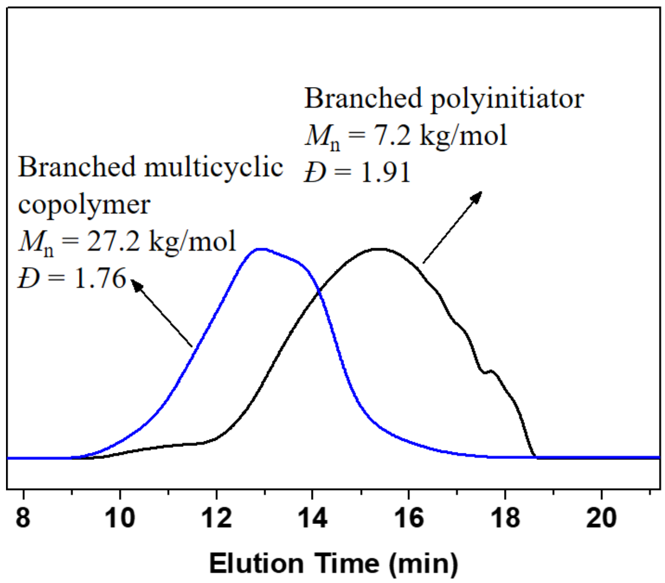

Supplementary Figure 43. SEC curves of resulting hyperbranched polyrhodanine initiator and hyperbranched multicyclic polymers

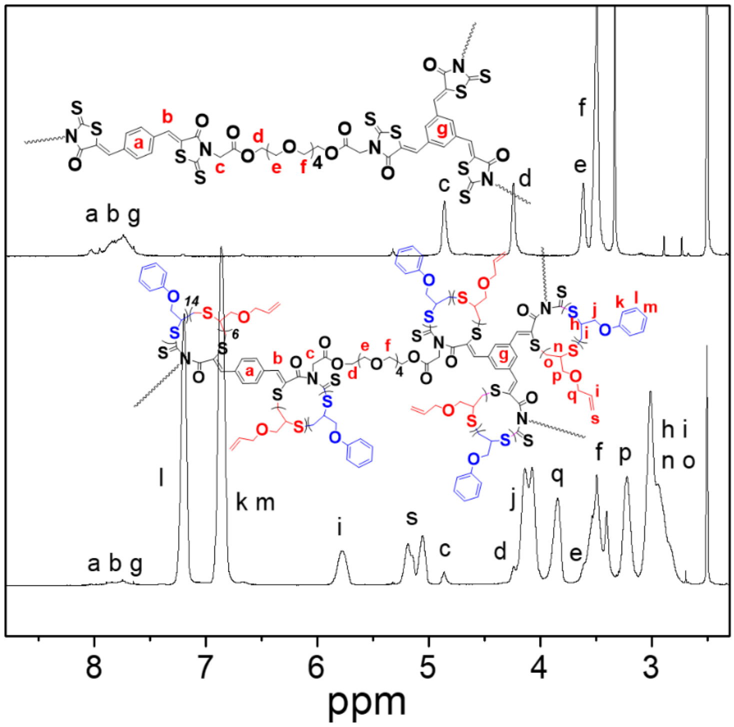

Supplementary Figure 44. ^{1}H NMR spectra of resulting hyperbranched polyrhodanine initiator and hyperbranched multicyclic copolymer

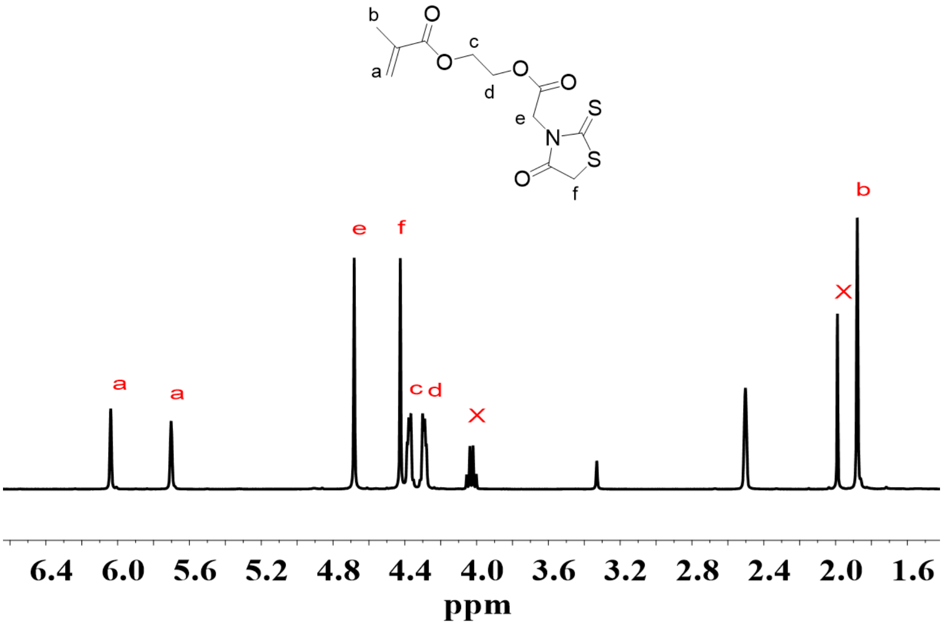

Supplementary Figure 45. ^1H NMR spectrum of rhodanine-containing methyl methacrylate (MRDA)

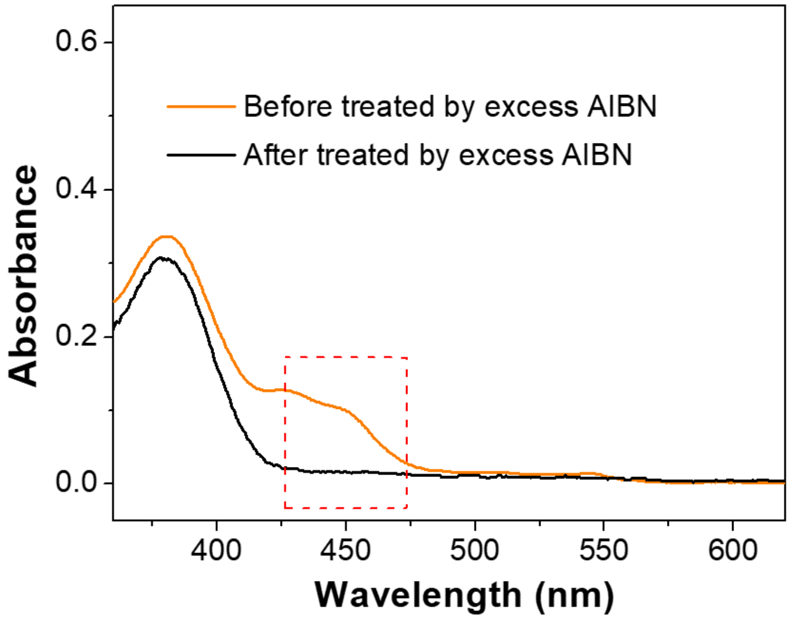

Supplementary Figure 46. UV−vis characteristic absorption curves of trithiocarbonate containing copolymer 1 and copolymer 2 in Figure 8

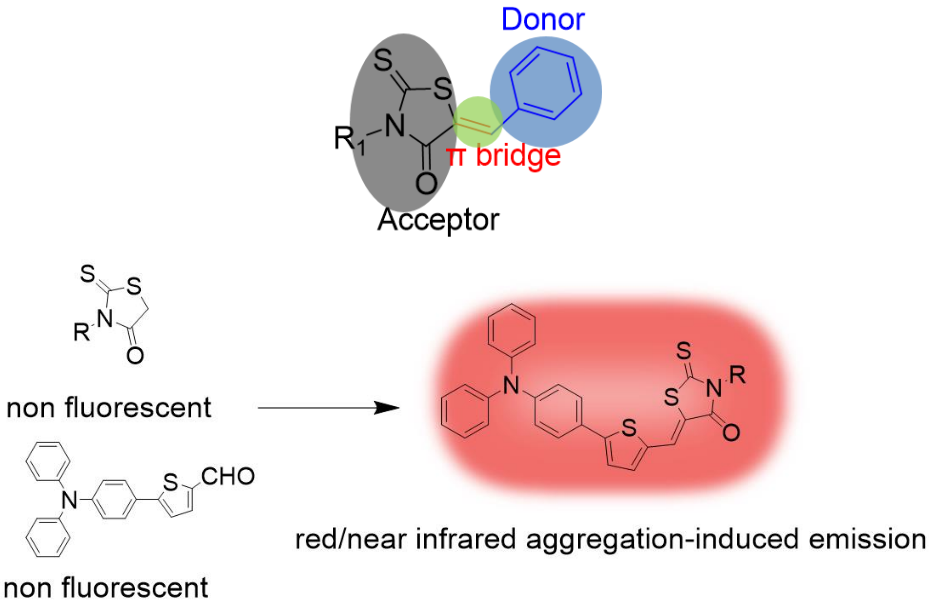

Supplementary Figure 47. The typical donor-acceptor structure of RA reaction product

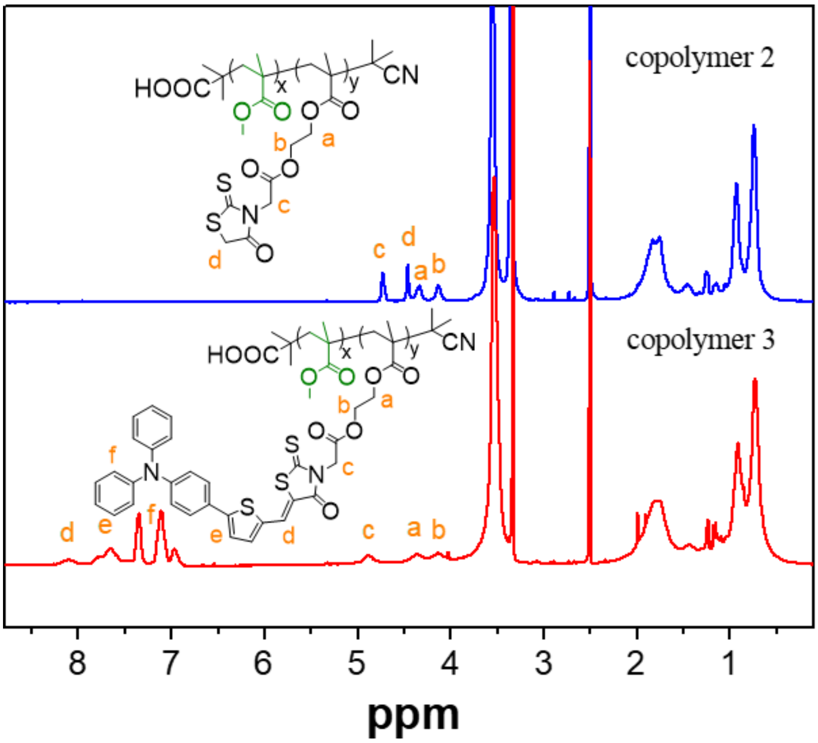

Supplementary Figure 48. The ^{1}H NMR spectra of copolymer 2 with side rhodanine units and red/near-infrared AIE copolymer 3 with side rhodanine units

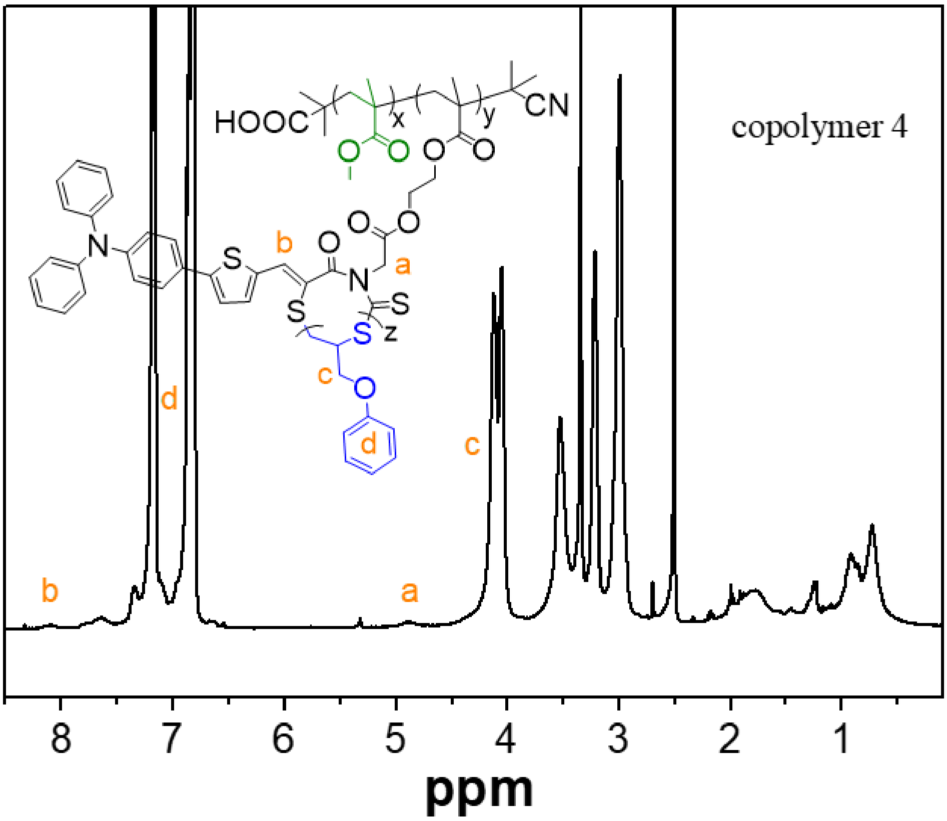

Supplementary Figure 49. The ^{1}H NMR spectrum of red/near-infrared AIE multicyclic polymer 4 with pendant cyclic units
